# Dual Cytoplasmic and Chloroplastic Mechanisms Fine‐Tune Chloroplast Division through ARC3 Protein Stability

**DOI:** 10.1002/advs.202523660

**Published:** 2026-05-26

**Authors:** Yang Yuan, Denghao Xiang, Ruiying Li, Zhichuang Yue, Zhipeng Wang, Honghong Hu

**Affiliations:** ^1^ National Key Laboratory of Crop Genetic Improvement Hubei Hongshan Laboratory Huazhong Agricultural University Wuhan China

**Keywords:** ARC3 stability, Chloroplast division, chloroplast protease, protein homeostasis, ubiquitin‐proteasome

## Abstract

Chloroplast division is essential for plant growth and photosynthesis, and the assembly of the FtsZ‐ring is tightly regulated by ARC3 (ACCUMULATION AND REPLICATION OF CHLOROPLASTS 3). However, the mechanisms controlling its protein turnover remain poorly understood. This study reveals that ARC3 stability is co‐regulated by both the ubiquitin‐proteasome system (UPS) and chloroplast protease pathways. We demonstrate that the E3 ubiquitin ligase PUB52 mediates cytosolic degradation of ARC3 precursors via the 26S proteasome, whereas the chloroplast chaperone‐protease CLPC1 (CLPC HOMOLOGUE 1) promotes ARC3 degradation within chloroplasts. Conversely, ARC2 stabilizes ARC3 by protecting it from proteolytic degradation, ensuring proper FtsZ‐ring assembly. Either disruption or overexpression of *PUB52*, *CLPC1*, or *ARC2* leads to aberrant chloroplast division phenotypes that resemble those of *ARC3* dysregulation mutants. Genetic analyses place these regulators upstream of ARC3 in the chloroplast division pathway. These findings establish a post‐translational regulatory network where PUB52, CLPC1, and ARC2 dynamically control ARC3 levels to fine‐tune chloroplast division. This work provides important insights into the coordination of cytoplasmic and chloroplast protein homeostasis systems in maintaining organellar homeostasis, with broader implications for organelle biogenesis and plant stress adaptation.

## Graphical abstract

ARC3 levels are controlled by cytosolic and chloroplast proteolytic systems. PUB52 mediates ARC3 precursor ubiquitination and degradation in the cytosol, while CLPC1 promotes ARC3 degradation in chloroplasts, where ARC2 protects ARC3 from excessive breakdown. Disrupting these components causes chloroplast division defects, placing them upstream of ARC3. This reveals a post‐translational network that precisely modulates chloroplast division, linking cytoplasmic and chloroplast proteostasis.

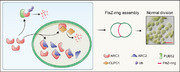

## 1 Introduction

Chloroplasts, originating from endosymbiosis, are crucial for photosynthesis and essential metabolic processes in plants [1, 2]. Due to their endosymbiotic origin, chloroplasts possess distinct genomes and replicate through the binary fission of pre‐existing plastids [3, 4, 5]. Chloroplast division is a highly conserved process across both cyanobacteria and eukaryotes, involving multiple well‐characterized regulatory components [6]. A critical initial step in this process is the assembly of the FtsZ ring (Z ring), which forms the division complex [7, 8]. Proteins localized within the inner and outer envelope membranes orchestrate the assembly and constriction of ring‐like structures spanning both membranes at the division site. Disruptions in regulatory machinery, particularly in Z ring formation, often result in altered chloroplast numbers and size [9, 10, 11, 12], leading to leaf chlorosis, growth retardation, and substantial biomass reductions [13, 14]. These findings underscore the importance of maintaining proper chloroplast morphology and quantity for efficient photosynthesis as well as normal plant growth.

FtsZ proteins are central components of chloroplast division, containing a conserved GTP‐binding domain at their N‐terminus and belonging to the tubulin‐like cytoskeletal GTPase family, which evolved from cyanobacterial endosymbiosis [8, 15]. While most bacteria have a single *FtsZ* gene encoding a protein that forms a contractile Z‐ring during cell division [16], most photosynthetic eukaryotes possess two *FtsZ* genes (*FtsZ1* and *FtsZ2*), which assemble into heteropolymer‐based Z‐rings at the chloroplast division site [7, 17]. The Z‐ring is highly dynamic, undergoing continuous subunit exchange via polymerization and depolymerization [18]. In *Arabidopsis*, FtsZ‐ring assembly is tightly regulated by several factors. ARC6 anchors FtsZ2 to the inner envelope membrane and promotes FtsZ protofilament assembly via direct interaction [19, 20]. The Min system (Minicell system) regulates both FtsZ self‐assembly and Z‐ring positioning, restricting its formation to mid‐plastid locations to prevent aberrant division [21, 22]. In bacteria, MinC inhibits Z‐ring assembly; however, in green‐lineage organisms, this function is assumed by ARC3, which primarily inhibits Z‐ring assembly at non‐division sites [12, 23]. *Arabidopsis* mutants lacking functional ARC3 show fewer, abnormally enlarged chloroplasts, with mislocalized parallel FtsZ rings. Conversely, overexpression of *ARC3* leads to punctate FtsZ filaments and chloroplast enlargement [11, 12]. A recent study reported that ARC3 accumulation declines during leaf expansion and plant development [24]. These findings highlight the necessity of precise regulation of ARC3 protein levels for proper chloroplast division in plants, yet the mechanisms governing this regulation remain unclear.

Chloroplast proteins are encoded by both nuclear and chloroplast genomes, necessitating a robust protein quality control system to maintain homeostasis during development and stresses [25, 26, 27]. The degradation of chloroplast proteins is mediated through three key mechanisms [28, 29]. The ubiquitin‐proteasome system (UPS) degrades various chloroplast proteins, including precursor proteins, envelope proteins, and internal plastid‐encoded proteins [30, 31]. For example, the E3 ligase CHIP (Carboxyl terminus of Hsc70‐Interacting Protein) specifically targets precursor proteins in the cytosol for UPS‐mediated degradation [32]. A chloroplast outer envelope‐localized RING‐type ubiquitin E3 ligase SP1 (Suppressor of PPI1 Locus 1) degrades TOC proteins under stress conditions to limit photosynthetic precursor protein import [33, 34]. The CDC48 complex (Cell Division Cycle 48) facilitates ubiquitin‐dependent proteostasis by targeting plastid‐encoded substrates, such as RbcL and AtpB [35]. Chloroplast protease systems, comprising ATP‐dependent proteases such as caseinolytic protease (Clp), filamentous temperature‐sensitive H (FtsH), and Lon protease, along with chaperones, collaborate to refold or degrade misfolded proteins [29, 36, 37]. The chaperone CLPC1, for instance, interacts with inner envelope transporters to facilitate substrate processing [38, 39]. Chloroplast autophagy selectively clears damaged chloroplasts, particularly those damaged by ROS [40]. During oxidative stress, the U‐box E3 ligase PUB4 (PLANT U‐BOX 4) ubiquitinates chloroplast proteins to target them for vacuole degradation [41]. Given that mutations or overexpression of *ARC3* disrupt chloroplast division [11], it is evident that its protein levels must be tightly regulated. However, the specific degradation mechanisms controlling ARC3 turnover remain elusive.

In this study, we demonstrated that ARC3 degradation is mediated by both the ubiquitin‐proteasome system and chloroplast protease pathways during chloroplast division. PUB52 promotes UPS‐dependent degradation of ARC3 precursors in the cytosol, while CLPC1 facilitates intra‐chloroplast ARC3 degradation via protease activity. ARC2 stabilizes ARC3 through direct interaction. These regulatory mechanisms fine‐tune FtsZ ring assembly and localization, ensuring proper chloroplast division. Our findings reveal a post‐translational regulatory network that dynamically controls ARC3 levels, providing new insights into chloroplast division regulation.

## 2 Results

### 2.1 ARC3 Protein Level Is Controlled by Both Proteasomal and Proteolytic Systems

**FIGURE 1:**
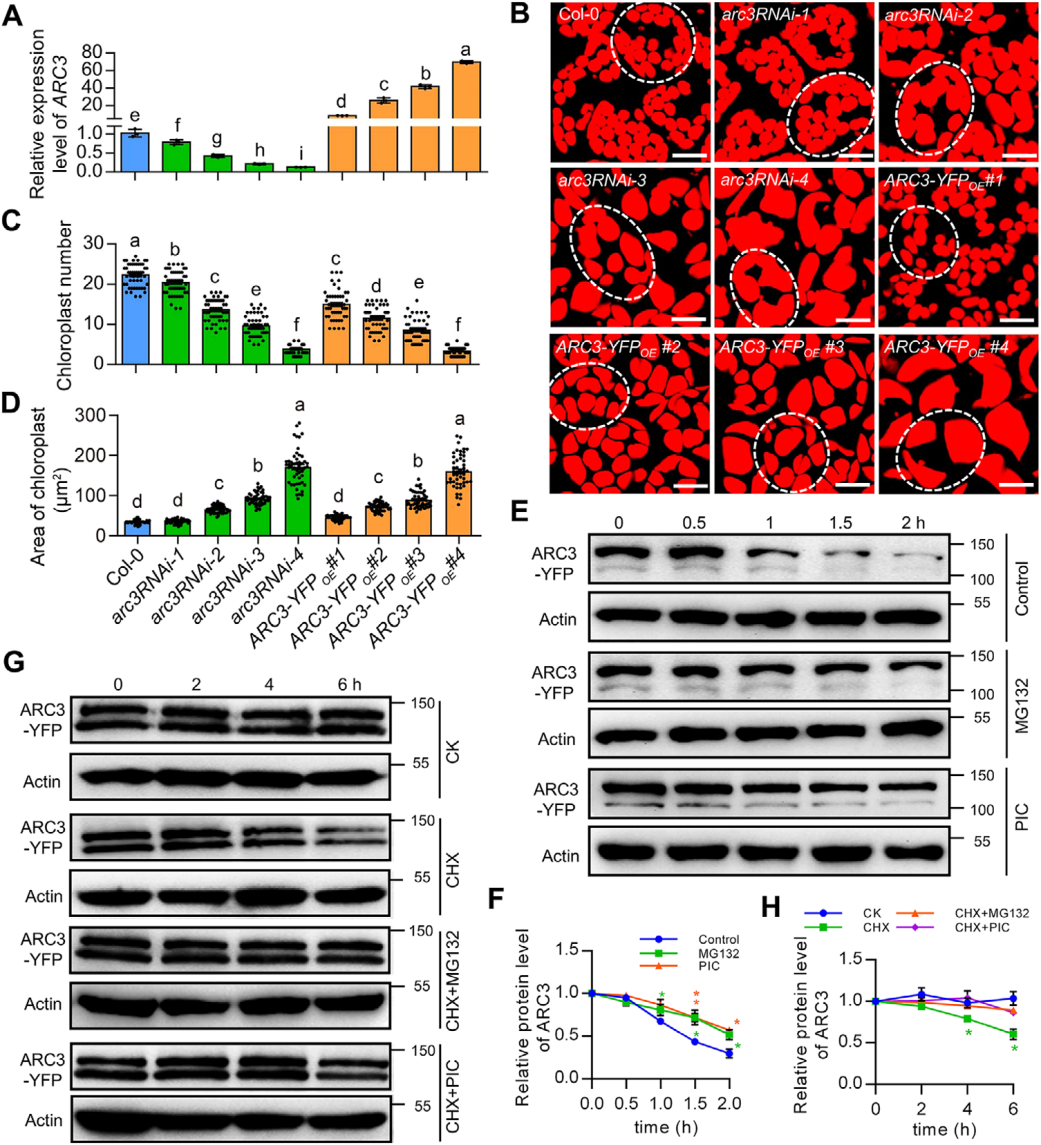
ARC3 is degraded by UPS proteasome and protease systems. (A) Quantitative PCR analyses of *ARC3* expression levels in 4‐week‐old Col‐0, *ARC3 RNAi* mutants (*arc3RNAi*), and *ARC3‐YFP* overexpression plants (*ARC3‐YFP_OE_*). Expression levels were normalized to *EF1α*. Data are means ± SEM (*n* = 3 independent experiments). (B) Observation of chloroplasts in 4‐week‐old plants in (A) through confocal microscopy. Dotted circles indicate mesophyll cells. Scale bar = 20 µm. (C, D) Quantitative analysis of chloroplast numbers in a plane (C) and chloroplast area (D) of Col‐0, *arc3RNAi* mutants, and *ARC3‐YFP_OE_* plants. Data presented are means ± SEM (three biological replicates, each with 50 cells per genotype). Different letters above error bars indicate a significant difference at *p* < 0.05, using one‐way ANOVA with Tukey's test. (E) ARC3 protein levels in *ARC3‐YFP* stably expressing plants (*ARC3‐YFP_OE_#1*) treated with proteasome inhibitor MG132 (middle panel) or protease inhibitor cocktail (PIC) (bottom panel) or not (control; upper panel) at 21°C for 2 h. Plant actin was used as a loading control for total protein extractions. (F) Relative fold changes of ARC3‐YFP to loading controls were quantified by ImageJ for (E). Data are means ± SEM (*n* = 3 independent experiments). Statistical analyses were performed using Student's *t*‐test. ^*^, *p* < 0.05; ^**^, *p* < 0.01. (G) ARC3 protein levels in *ARC3‐YFP* stably expressing plants (*ARC3‐YFP_OE_#1*) treated with cycloheximide (CHX) (middle panel) or CHX with MG132 (lower panel) or CHX with protease inhibitor cocktail (PIC) (bottom panel) or not (CK; upper panel) at 21°C for 6 h. Plant actin was used as a loading control for total protein extractions. (H) Relative fold changes of ARC3‐YFP to loading controls were quantified by ImageJ for (G). Data are means ± SEM (*n* = 3 independent experiments). Statistical analyses were performed using Student's *t‐*test. ^*^, *p* < 0.05. The original blot can be found in Figure S10.

To systematically investigate the impact of ARC3 threshold levels on chloroplast division in *Arabidopsis*, we generated *ARC3* knockdown (*arc3RNAi*) lines via RNAi technology and created YFP‐tagged *ARC3* overexpression plants (*ARC3‐YFP_OE_*), which successfully complemented the chloroplast division phenotype of the *arc3‐2* mutant (Figure S1). We analyzed the chloroplast division phenotypes of four lines with increased *ARC3* expression and four lines with decreased expression (Figure 1A). Our findings showed that slight up‐regulation or down‐regulation of *ARC3* resulted in a decrease in chloroplast number, while not significantly affecting their size. However, modulation to high levels of *ARC3* suppression or overexpression significantly impaired chloroplast division, resulting in fewer but larger chloroplasts (Figure 1B–D), consistent with previous reports [11, 42]. The severity of the observed phenotypes directly correlated with ARC3 levels, indicating a dose‐dependent correlation between ARC3 abundance and chloroplast division defects, consistent with prior findings [11].

To elucidate the mechanisms controlling ARC3 protein levels, we performed time‐course degradation assays using total protein extracts from *ARC3‐YFP_OE_* plants treated with MG132 (a proteasome inhibitor) or PIC (protease inhibitor cocktail). Under control conditions, ARC3 exhibited progressive degradation, whereas treatment with MG132 or PIC significantly slowed its turnover and stabilized the protein (Figure 1E,F), indicating dual regulation of ARC3 levels by both proteasomal and proteolytic systems. To further investigate whether the stability of the ARC3 protein in plants is regulated by these two pathways, we treated two‐week‐old seedlings grown on 1/2 MS medium with the protein synthesis inhibitor cycloheximide (CHX). Protein samples were collected at various time points post‐treatment. ARC3 levels declined progressively in CHX‐treated seedlings, but this degradation was markedly suppressed upon co‐treatment with either MG132 or PIC. Without CHX treatment, ARC3 levels remained stable throughout the time course (Figure 1G,H). These findings demonstrate that ARC3 stability is regulated by the ubiquitin‐proteasome (UPS) and proteolytic system.

Interestingly, in *ARC3‐YFP_OE_* transgenic plants, we detected not only the expected ∼112 kDa band but also an additional band approximately 15 kDa smaller. The relative intensities of these bands varied across experiments. Given that ARC3 protein contains ten cysteine residues, we hypothesized that the smaller band might represent an oxidized form resulting from disulfide bond formation. To test this, we mutated nine (ARC3^9C‐S^, Cys 42, 51, 54, 65, 93, 207, 299, 314, 611) or all ten (ARC3^10C‐S^) of cysteines into serine residues. Western blot analysis revealed the absence of the smaller band in both variants (Figure S1A). When expressed in *E. coli* without the transit peptide, the smaller band persisted, strongly supporting its identity as an oxidized state. Interestingly, we found that the ARC3 protein level declined gradually during leaf senescence, accompanied by a marked elevation in the ratio of its oxidized state to reduced state (Figure S1B). Moreover, expressing *ARC3^9^* ^C‐S^ *‐YFP* in *arc3*‐*2* mutant plants fully rescued the phenotype of fewer and larger chloroplasts (Figure S1C), confirming that ARC3 functions in chloroplast division primarily in a reduced state.

### 2.2 ARC3 Interacts with PUB52, CLPC1, and ARC2

**FIGURE 2:**
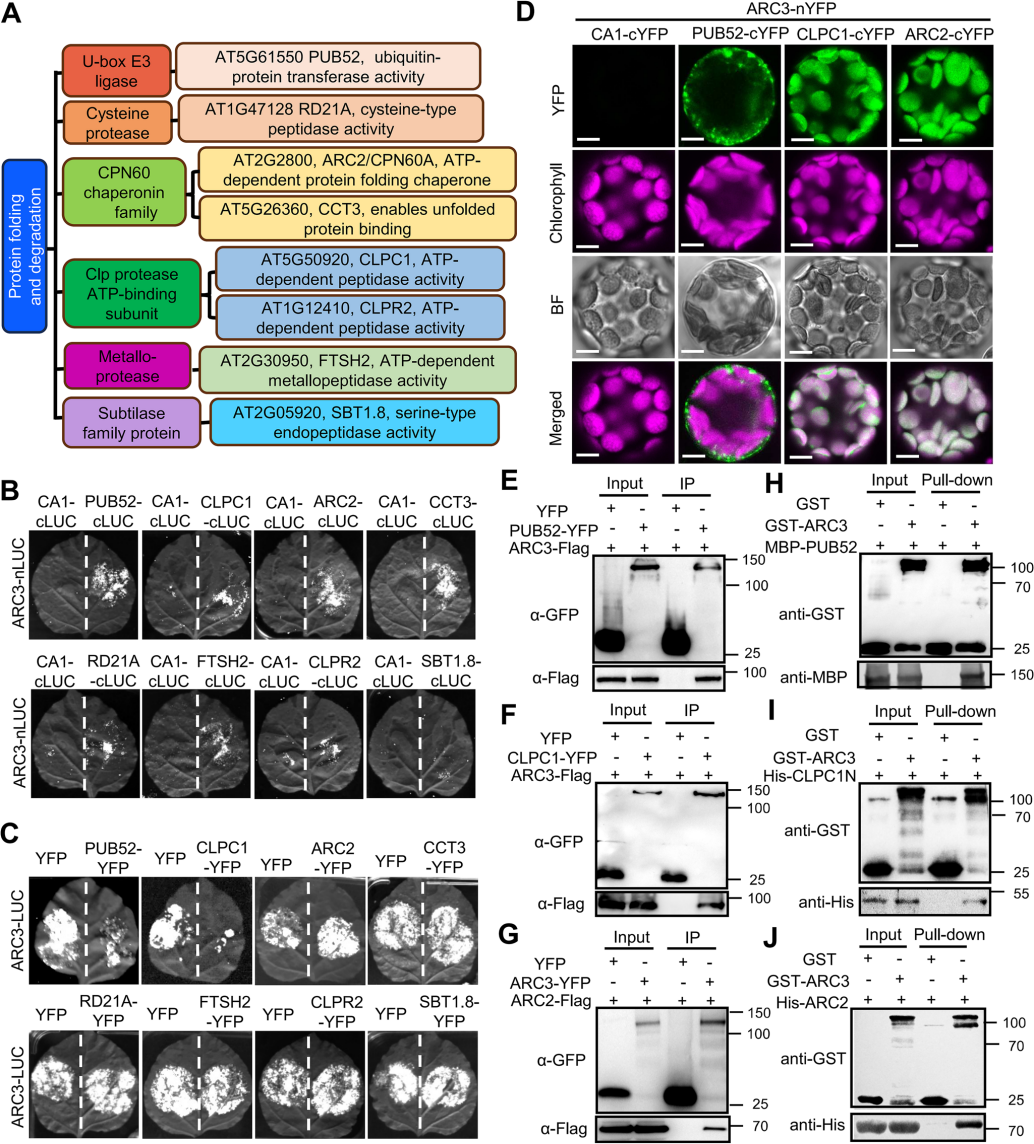
ARC3 interacts with PUB52, CLPC1, and ARC2 in vivo and vitro. (A) Potential ARC3 interactors belonging to the protein folding and degradation process from IP‐MS analysis. (B) The interactions of ARC3 with putative ARC3 interactors belonging to the protein folding and degradation process by split‐luciferase (split‐LUC) assays in *Nicotiana benthamiana* leaves. CA1 (BETA‐CARBONIC ANHYDRASE 1) served as a negative control. (C) The effect of ARC3 interactors on ARC3 protein stability in *N. benthamiana* leaves. YFP was used as a control. ARC3‐LUC was co‐expressed with YFP‐tagged ARC3 interactors or YFP alone in *N. benthamiana* leaves. (D) The interactions of ARC3 with PUB52, CLPC1, and ARC2 were studied using bimolecular fluorescence complementation (BiFC) assays in Arabidopsis protoplasts. CA1‐cYFP was used as a negative control. BF, bright field. Scale bar = 10 µm. (E–G) In vivo interactions of ARC3 with PUB52 (E), CLPC1 (F), and ARC2 (G) in stable transgenic Arabidopsis plants by coimmunoprecipitation (Co‐IP) assays. Total protein extracts from the plants in (E–G) were used as input. Input proteins were immunoprecipitated with GFP‐beads. The input and co‐immunoprecipitated proteins were detected with anti‐GFP and anti‐Flag antibodies as indicated. (H–J) Physical interactions of ARC3 with PUB52 (H), CLPC1 (I), and ARC2 (J) by pull‐down assays. MBP‐PUB52, His‐CLPC1N (1‐252 AA), His‐ARC2, and GST‐ARC3 were expressed in *E. coli* strain BL21 (DE3). The input and pulled‐down proteins were detected with anti‐GST, anti‐His or anti‐MBP antibodies as indicated. The original blot can be found in Figure S10.

To elucidate the regulatory mechanisms governing ARC3 stability *in planta*, we performed immunoprecipitation‐mass spectrometry (IP‐MS) on GFP‐affinity purified complexes from *ARC3‐YFP* overexpression lines. This proteomic profiling identified 220 high‐confidence interactors, which were classified into 17 biological processes via DAVID enrichment analyses. These processes included secondary metabolite biosynthesis, stress responses, and proteostasis regulation (protein folding/degradation) (Figure S2A). Notably, within the pathways associated with protein folding/degradation, several key proteins were identified: PUB52 in the proteasomal pathway; RD21A, CLPC1, CLPR2, FTSH2, and SBT1.8 in the proteolytic pathway; as well as ARC2 and CCT3 belonging to the CPN60 chaperonin family (Figure 2A). This interactome suggests a coordinated regulation of ARC3 levels through distinct cytoplasmic and chloroplast proteostasis systems.

Split‐luciferase complementation assays in *Nicotiana benthamiana* (*N. benthamiana*) confirmed robust interactions of ARC3 with PUB52, CLPC1, ARC2, CCT3, FTSH2, and RD21A (Figure 2B). We further examined which proteins influence ARC3‐LUC stability in *N. benthamiana*. Co‐expression of PUB52‐YFP or CLPC1‐YFP attenuated the luminescence signal from ARC3‐LUC, conversely, co‐expression of ARC2‐YFP enhanced signal intensity (Figure 2C; Figure S2B–I). Other tested proteins showed no effects on ARC3 stability. These findings suggest that PUB52 and CLPC1 may promote degradation of the ARC3 protein, whereas ARC2 appears to stabilize it.

We further validated the interactions between ARC3 and its interactors─PUB52, CLPC1, and ARC2─by bimolecular fluorescence complementation (BiFC) assays in *Arabidopsis* protoplasts. ARC3 interacted with PUB52 in the cytoplasm, and with CLPC1 and ARC2 in chloroplasts (Figure 2D), consistent with the subcellular localizations observed in stable transgenic plants: PUB52 localized to the cytoplasm, CLPC1 and ARC2 localized to the chloroplast, while ARC3 was present in both compartments (Figure S3). To biochemically confirm these interactions, we performed co‐immunoprecipitation (Co‐IP) assays using stable transgenic plants co‐expressing *35S:PUB52‐YFP* and *35S:ARC3‐Flag*, *35S:CLPC1‐YFP* and *35S:ARC3‐Flag*, or *35S:ARC2‐Flag* and *35S:ARC3‐YFP*. The results confirmed that ARC3‐Flag was co‐immunoprecipitated with both PUB52‐YFP and CLPC1‐YFP; moreover, PUB52 specifically immunoprecipitated ARC3 from cytosolic fractions but not from chloroplast fractions (Figure 2E,F; Figure S3D), and ARC2‐Flag was co‑immunoprecipitated with ARC3‐YFP (Figure 2G). To determine if these interactions were direct, we next conducted pull‐down assays with recombinant proteins expressed in *E. coli*. GST‐ARC3 pulled down MBP‐PUB52, His‐ARC2, and a truncated version of His‐CLPC1N (1‐252 AA, used due to difficulties in expressing full‐length CLPC1), but not GST alone (Figure 2H–J). Collectively, these in vivo and in vitro findings demonstrate that ARC3 physically interacts with PUB52, CLPC1, and ARC2, establishing a regulatory network that controls ARC3 stability.

### 2.3 PUB52 and CLPC1 Are Involved in Chloroplast Division

While ARC2 has a known role in chloroplast division, the specific roles of *PUB52* and *CLPC1* in this process remain unclear. To address this, we generated both overexpression (YFP‐tagged) and knock‐out mutants of *PUB52* and *CLPC1* using CRISPR/Cas9 system [43]. For *PUB52* gene, we obtained two mutant alleles: one with an 829‐bp deletion (starting at position 2271^st^ bp) and another with a 1‐bp deletion (at 2033^rd^ bp), resulting in two truncated proteins: *pub52‐c1* (578 amino acids) and *pub52‐c2* (536 AA) (Figure S4A). Similarly, for *CLPC1*, we identified a 2‐bp insertion (*clpc1‐c1*)─one at 12^th^ bp and another at 16^th^ bp, and a 4‐bp deletion starting at 1051^st^ bp (*clpc1‐c2*). These alterations resulted in premature termination at the 15^th^ and 239^th^ amino acids, respectively (Figure S4C).

**FIGURE 3:**
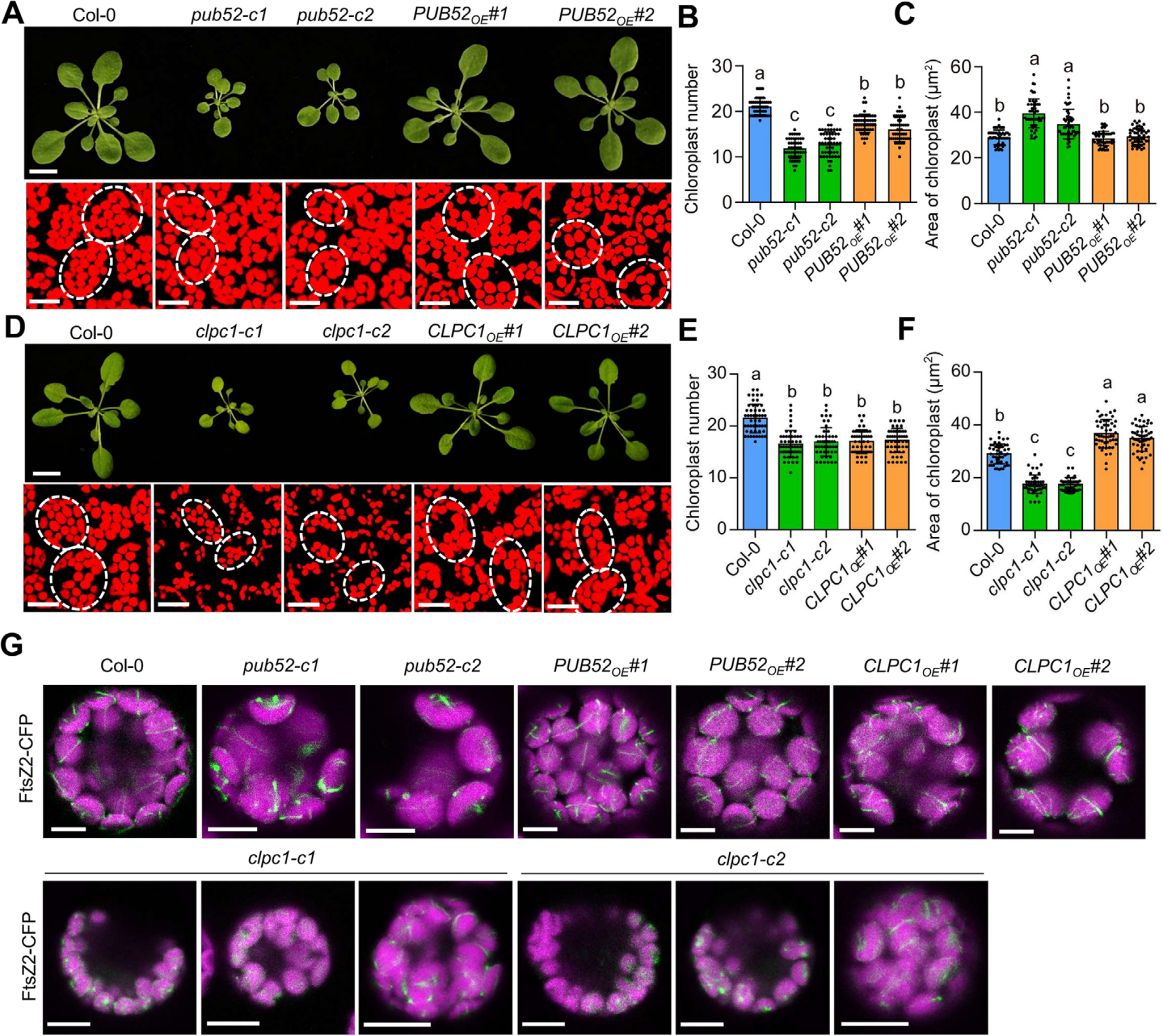
PUB52 and CLPC1 are involved in chloroplast division. (A) The phenotypes of growth (upper panels, scale bar = 1 cm) and chloroplasts (bottom panels, scale bar = 20 µm) of 4‐week‐old Col‐0, *PUB52 crispr* mutants (*pub52‐c*) and *PUB52‐YFP* overexpressing plants (*PUB52_OE_*). Dotted circles indicate mesophyll cells. (B, C) Quantitative analysis of chloroplast numbers per cell in a plane (B) and chloroplast size (C) of the indicated genotypes. (D) The phenotype of plants (upper panels, scale bar = 1 cm) and chloroplasts (bottom panels, scale bar = 20 µm) of 4‐week‐old Col‐0, *clpc1‐crispr* mutants (*clpc1‐c*) and *CLPC1_OE_* (*CLPC1‐YFP* overexpression) plants. (E, F) Quantitative analysis of chloroplast numbers per cell in a plane (E) and chloroplast size (F) of the indicated genotypes. Dotted circles indicate mesophyll cells. Data presented are means ± SEM (three biological replicates, each with 50 cells per genotype). Different letters above error bars indicate a significant difference at *p* < 0.05, using one‐way ANOVA with Tukey's test. (G) Localization patterns of FtsZ2‐CFP fusion proteins transiently expressed in protoplasts isolated from 4‐week‐old Col‐0, *pub52‐c*, *PUB52_OE_*, *clpc1‐c*, and *CLPC1_OE_* plants. Fluorescent FtsZ2‐CFP and chlorophyll signals were in green and magenta, respectively. Merged images are shown. Scale bar = 10 µm.

Notably, while *PUB52_OE_* and *CLPC1_OE_* plants exhibited rosette sizes comparable to Col‐0, the *pub52‐c* mutants showed reduced size, and *clpc1‐c* mutants displayed obvious chlorosis and growth retardation (Figure 3A,D; Figure S4B,D). To determine the role of PUB52 and CLPC1 in chloroplast division, we examined chloroplast phenotypes using confocal microscopy. Both *pub52‐c* mutants and *PUB52_OE_* plants, as well as *clpc1‐c* and *CLPC1_OE_* plants, showed a significant reduction in chloroplast number (Figure 3A,B,D,E). Chloroplasts in *pub52‐c* mutants and *CLPC1_OE_* plants were larger than those observed in Col‐0, while *PUB52_OE_* plants maintained chloroplast sizes comparable to Col‐0. Interestingly, *clpc1‐c* mutants exhibited smaller chloroplasts than Col‐0 (Figure 3C, F), likely due to the role of CLPC1 in maintaining proteome and RNA homeostasis [38]. These findings underscore the critical roles of PUB52 and CLPC1 in chloroplast division.

The assembly of the FtsZ ring at the mid‐division site is essential for proper chloroplast division. We assessed FtsZ2 assembly by transiently expressing *FtsZ2‐CFP* in protoplasts from these lines. RT‐PCR showed low *CFP* expression in the *clpc1* mutant compared to the wild type, with no significant differences among other genotypes, indicating comparable *FtsZ2‐CFP* expression efficiency across all backgrounds except *clpc1* (Figure S4E). Imaging and statistical analysis of FtsZ ring assembly revealed that in Col‐0 and *PUB52_OE_* chloroplasts, complete FtsZ rings mostly formed at the division site. However, in *pub52‐c* mutants, FtsZ2 failed to assemble into rings and instead appeared as discrete dots or short filaments. In *CLPC1_OE_* chloroplasts, no clear Z‐rings formed at the division site; instead, multiple aberrant Z‐rings formed at ectopic locations (Figure 3G; Figure S4F). In *clpc1* mutants, weak fluorescence likely reflects low transformation efficiency of the *FtsZ2‐CFP* construct and severe chloroplast developmental defects; quantitative analysis confirmed most FtsZ2 localized diffusely in the stroma, with discernible Z‐rings in only approximately 30–40% of cells (Figure 3G; Figure S4F). These FtsZ2 assembly defects in *pub52* mutant and *CLPC1* genetic materials resemble those seen in *ARC3* overexpression and loss‐of‐function mutants, strongly suggesting that PUB52 and CLPC1 regulate chloroplast division by modulating ARC3 protein levels.

### 2.4 PUB52 Regulates Chloroplast Division by Direct Ubiquitination and Degradation of ARC3

**FIGURE 4:**
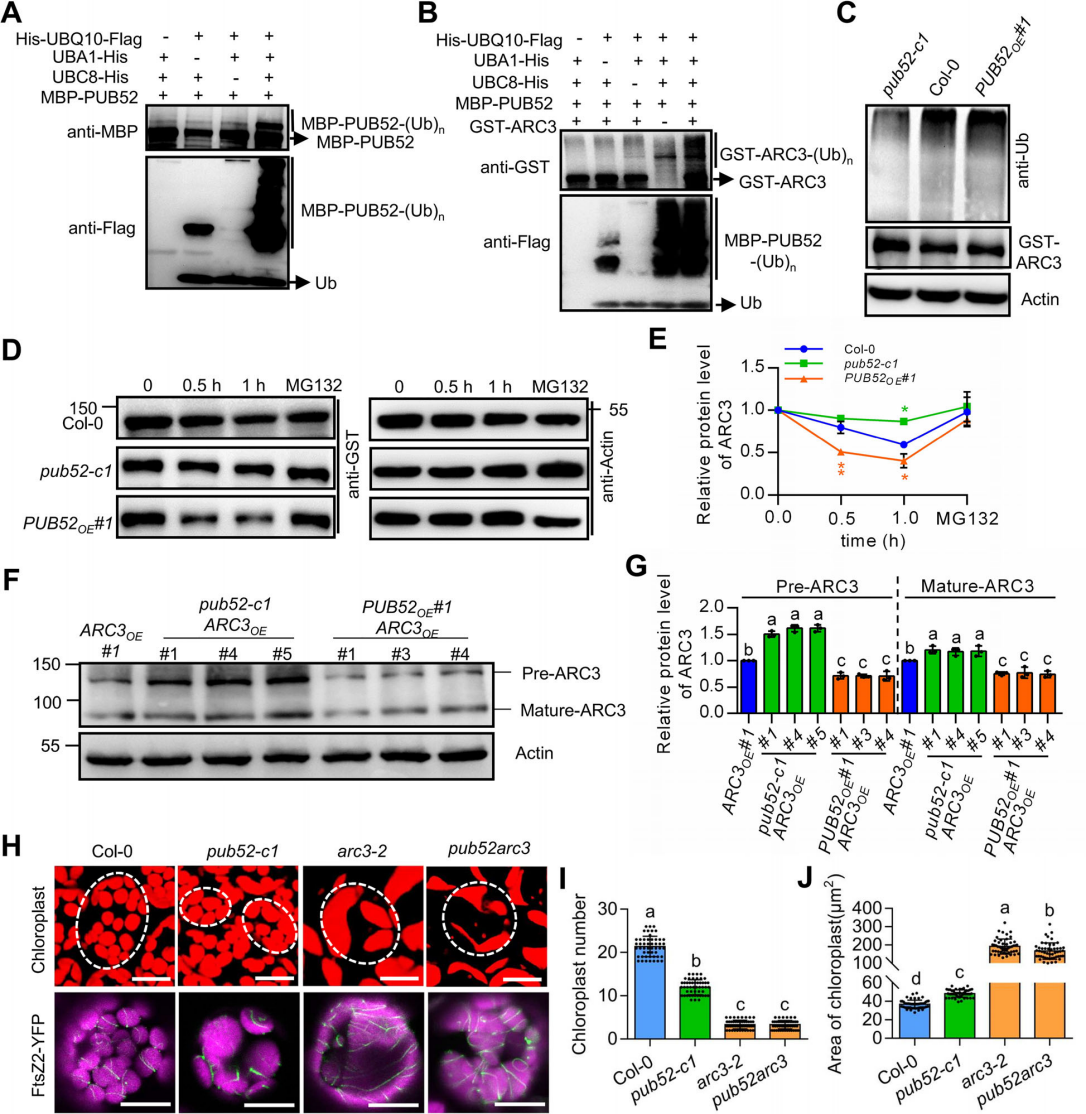
PUB52 ubiquitinates ARC3 and modulates its abundance. (A) In vitro ubiquitination assays of PUB52 with E1 (His‐UBA1), E2 (His‐UBC8), ubiquitin (His‐UBQ10‐Flag), and ATP or not. Immunoblotting was analyzed with anti‐MBP and anti‐Flag antibodies. (B) In vitro ubiquitination of PUB52 on ARC3. MBP‐PUB52 and GST‐ARC3 were incubated with E1, E2, ubiquitin, and ATP or not. ARC3 polyubiquitination was detected with anti‐GST antibody. (C) Semi‐in vitro ubiquitination assays of GST‐ARC3 proteins with protein extracts from 4‐week‐old Col‐0, *pub52‐c1* or *PUB52_OE_#1* plants. Immunoblotting was analyzed with anti‐Ub, anti‐GST, and anti‐Actin antibodies. (D) Cell‐free degradation assay of ARC3 degradation by PUB52. Purified GST‐ARC3 was incubated with protein extracts from 4‐week‐old Col‐0, *pub52‐c1* or *PUB52_OE_#1* plants at 22°C with or without MG132. MG132 indicates samples collected after 1 hour of MG132 treatment. Actin was used as the internal control. (E) Relative fold changes of ARC3‐GST to loading controls were quantified by ImageJ for (D). Data are means ± SEM (*n* = 3 independent experiments). Statistical analyses were performed using Student's *t*‐test. ^*^, *p* < 0.05; ^**^, *p* < 0.01. (F) ARC3 protein levels in 4‐week‐old Col‐0, *pub52‐c1* and *PUB52_OE_#1* plants stably expressing *35S:ARC3‐Flag*. *ARC3_OE_* #1, *35S:ARC3‐Flag* line in Col‐0; *pub52‐c1 ARC3_OE_*, *35S:ARC3‐Flag* overexpression in *pub52‐c1*; *PUB52_OE_#1 ARC3_OE_*, *35S:ARC3‐Flag* overexpression in *PUB52_OE_#1*. Plant actin was used as a loading control for total protein extractions. (G) Quantitative analysis of relative ARC3 protein (Pre‐protein and Mature‐protein) level of the indicated genotypes in (F). Data presented were means ± SEM (three biological replicates). Different letters above error bars indicate a significant difference at *p* < 0.05, using one‐way ANOVA with Tukey's test. (H) The chloroplasts (upper panels) in 4‐week‐old Col‐0, *pub52‐c1*, *arc3‐2*, and *pub52arc3* leaves, and the localization patterns of FtsZ2‐YFP fusion proteins (bottom panels) were transiently expressed in their protoplasts. Fluorescent FtsZ2‐YFP and chlorophyll signals were shown in green and magenta, respectively. Merged images are shown. Scale bar = 20 µm. Dotted circles indicate mesophyll cells. (I, J) Quantitative analysis of chloroplast numbers per cell in a plane (I) and chloroplast size (J) of the indicated genotypes. Data are means ± SEM (three biological replicates, each with 50 cells per genotype). Different letters above error bars indicate a significant difference at *p* < 0.05, using one—way ANOVA with Tukey's test. The original blot can be found in Figure S11.

PUB52 is predicted to possess E3 ligase activity. To test this, we expressed recombinant MBP‐PUB52 proteins in *E. coli* for in vitro ubiquitination assays. In the presence of ubiquitin, ubiquitin‐activating enzyme (E1), and ubiquitin‐conjugating enzyme (E2), MBP‐PUB52 exhibited robust auto‐ubiquitination, whereas control reactions lacking any of the components showed no detectable activity (Figure 4A). Since PUB52 physically interacts with ARC3 (Figure 2D,E,H), we next examined whether PUB52 could mediate ARC3 ubiquitination. In vitro ubiquitination assays confirmed that GST‐ARC3 was efficiently ubiquitinated by PUB52 only in the presence of E1, E2, and ubiquitin (Figure 4B).

We further conducted a semi‐in vitro ubiquitination assay by incubating purified GST‐ARC3 with total protein extracts from 4‐week‐old Col‐0, *pub52‐c1*, and *PUB52_OE_ #1* leaves, supplemented with 50 µm MG132. These assays revealed enhanced polyubiquitination of ARC3 in *PUB52_OE_#1* extracts and reduced ubiquitination in *pub52‐c1* compared to Col‐0 (Figure 4C). Consistent with this, cell‐free degradation assays showed that GST‐ARC3 degradation was significantly slower in *pub52‐c1* extracts and faster in *PUB52_OE_#1* extracts relative to Col‐0. Degradation was effectively blocked by MG132 in all genotypes (Figure 4D,E), confirming proteasome dependence.

To assess these findings *in planta*, we expressed *35S:ARC3‐Flag* in Col‐0, *pub52‐c1*, and *PUB52_OE_#1* plants. When *ARC3* transcript levels were similar across genotypes (Figure S5A,B), ARC3‐Flag protein levels (both the precursor and mature forms) were significantly higher in *pub52‐c1* background and lower in *PUB52_OE_#1* background compared to Col‐0 background (*ARC3_OE_#1*), notably, this differential accumulation was more pronounced for the precursor form (Figure 4F,G), supporting a role for PUB52 in promoting proteasomal degradation of the ARC3 precursor via the 26S proteasome.

To investigate whether PUB52 regulates chloroplast division through ARC3, we generated a *pub52arc3* double mutant by crossing *pub52‐c1* and *arc3‐2*. The double mutant exhibited chloroplast morphology and size phenocopying the *arc3‐2* single mutant (Figure 4H–J). Furthermore, while the *pub52‐c1* single mutant showed short, disorganized FtsZ2 filaments, similar to *ARC3* overexpression plants as previously reported [11], the *pub52arc3* mutant displayed multiple mislocalized and elongated FtsZ filaments typical of *arc3‐2* plants (Figure 4H; Figure S5C,D). To further elucidate the functional relationship between *ARC3* and *PUB52*, we quantified chloroplast size and number in *PUB52_OE_#1* transgenic lines overexpressing *35S: ARC3‐Flag* (*PUB52_OE_#1 ARC3_OE_*). The degree to which chloroplast division defects observed in *PUB52_OE_#1* plants were rescued depended on the level of *ARC3* overexpression. Moderate *ARC3* overexpression (∼5‐fold, line #5) was sufficient to restore chloroplast number in *PUB52_OE_#1*. In contrast, higher *ARC3* expression levels (>10‐fold, lines #1, #3, #6) induced a chloroplast division phenotype resembling that of canonical *ARC3* overexpression lines (Figure S5E–G). Together, these biochemical and genetic findings demonstrate that PUB52 modulates chloroplast division via regulating ARC3 protein stability through ubiquitin‐mediated degradation.

### 2.5 CLPC1 Participates in Chloroplast Division by Regulating the Stability of ARC3 Protein

**FIGURE 5:**
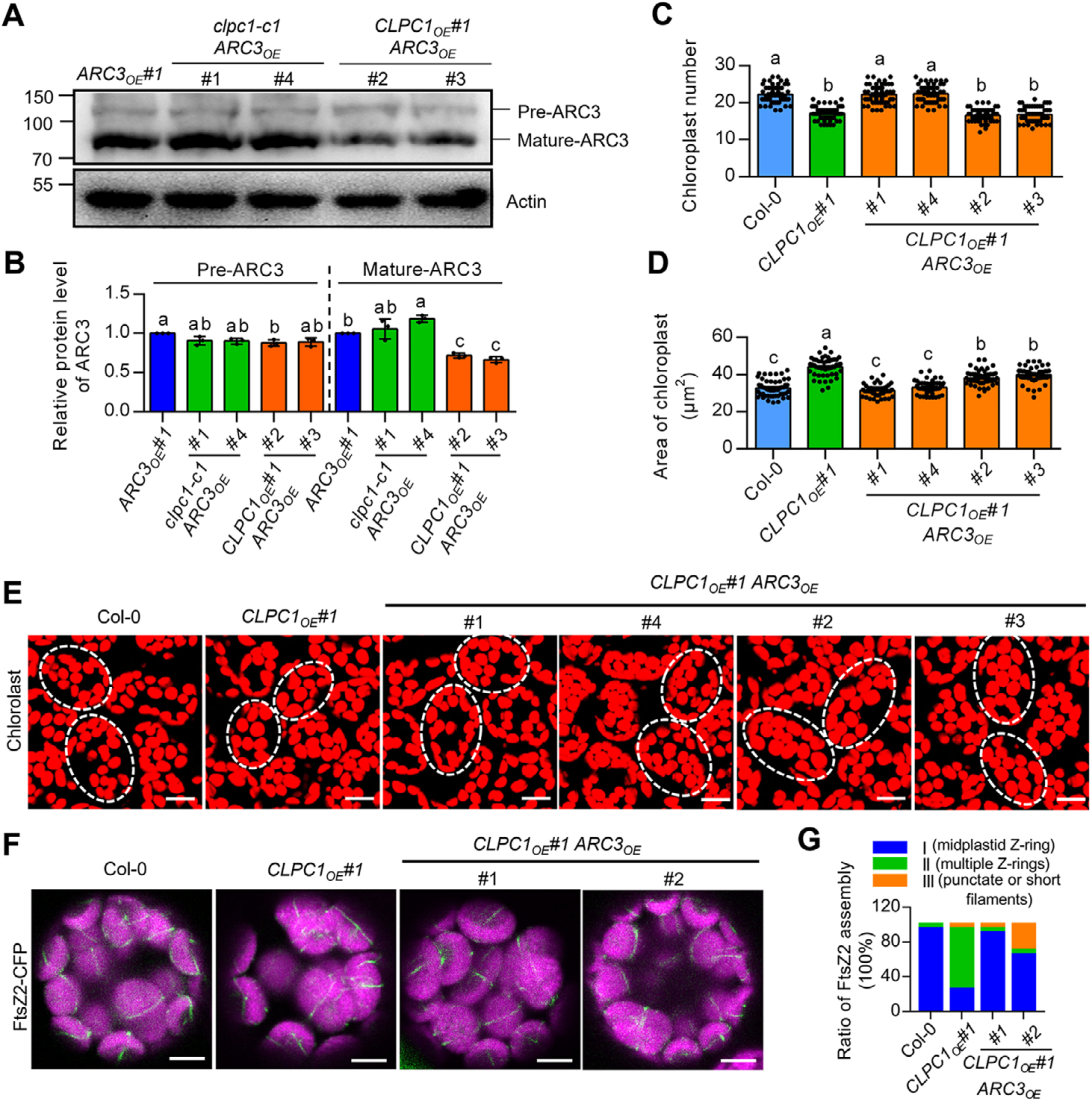
CLPC1 participates in chloroplast division by regulating the stability of ARC3 protein. (A) Protein levels in 4‐week‐old *ARC3_OE_#1*, *35S:ARC3‐Flag* overexpressing *clpc1‐c1* (*clpc1‐c1 ARC3_OE_*) and *CLPC1_OE_#1* (*CLPC1_OE_#1 ARC3_OE_*) lines with comparable *ARC3* expression levels. Plant actin was used as a loading control for total protein extractions. The original blot can be found in Figure S11. (B) Quantitative analysis of relative ARC3 protein (Pre‐protein and Mature‐protein) level of the indicated genotypes in (A). Data presented were means ± SEM (three biological replicates). Different letters above error bars indicate a significant difference at *p* < 0.05, using one‐way ANOVA with Tukey's test. (C, D) Quantitative analysis of chloroplast numbers per cell in a plane (C) and chloroplast area (D) of 4 ‐week ‐old Col‐0, *CLPC1_OE_#1*, and *CLPC1_OE_#1* overexpressing *35S:ARC3‐Flag* lines (*CLPC1_OE_#1 ARC3_OE_*). Data presented are means ± SEM (three biological replicates, each with 50 cells per genotype). Different letters above error bars indicate a significant difference at *p* < 0.05, using one‐way ANOVA with Tukey's test. (E) The mesophyll cell chloroplasts in the indicated genotypes through confocal microscopy. Dotted circles indicate mesophyll cells. Scale bar = 10 µm. (F) The localization patterns of FtsZ2‐CFP fusion proteins transiently expressing in 4‐week‐old Col‐0, *CLPC1_OE_#1*, and *CLPC1_OE_#1 ARC3_OE_* protoplasts. Fluorescent FtsZ2‐CFP and chlorophyll signals were shown in green and magenta, respectively. Merged images are shown. Scale bar = 10 µm. (G) Proportion of different assembly forms of FtsZ2 for each genotype in (F). Each with 20 cells per experiment.

To investigate whether CLPC1 regulates ARC3 stability in vivo, we expressed *35S:ARC3‐Flag* in *clpc1‐c1* and *CLPC1_OE_#1* plants. Despite comparable transcript levels of *ARC3* in these backgrounds (Figure S6A,B), the abundance of mature ARC3‐Flag protein was elevated in *clpc1‐c1* mutants and significantly decreased in *CLPC1_OE_#1* plants compared to *ARC3_OE_#1*, in contrast, the level of the ARC3 precursor form showed no significant difference (Figure 5A,B), confirming that CLPC1 modulates the intra ‐chloroplastic stability of ARC3 in *planta*.

Given the direct interaction between CLPC1 and ARC3, we asked whether CLPC1 affects chloroplast division through ARC3. We generated a *clpc1arc3* double mutant by crossing *clpc1‐c1* with *arc3‐2*. The double mutant phenocopied the *arc3‐2* single mutant, exhibiting fewer and enlarged chloroplasts (Figure S6C,D). To further clarify their functional relationship, we quantified chloroplast size and number in *CLPC1_OE_#1* transgenic lines overexpressing *35S*:*ARC3‐Flag* (designated *CLPC1_OE_#1 ARC3_OE_*). *ARC3* overexpression at varying levels differentially rescued the chloroplast division defects observed in *CLPC1_OE_#1* plants. Moderate overexpression (about 5‐fold, lines #1 and #4) substantially restored chloroplast division, whereas higher overexpression resulted in a less pronounced rescue (10‐fold, lines #2 and #3) (Figure 5C–E; Figure S6B), consistent with the ARC3 role in chloroplast division. We further observed FtsZ‐ring assembly and found that *CLPC1_OE_#1* chloroplasts contained elongated FtsZ2 filaments at ectopic sites. Moderate *ARC3* overexpression effectively restored the formation of normal midplastid Z‐rings, while higher ARC3 levels only partially rescued aberrant filament elongation and impaired midplastid Z‐ring formation (Figure 5F,G; Figure S6E). These findings demonstrate that *ARC3* overexpression compensates for FtsZ misassembly in *CLPC1_OE_#1* plants in a dose‐dependent manner, indicating that CLPC1 functions primarily through ARC3 to regulate chloroplast division.

### 2.6 ARC2 Regulates Chloroplast Division by Protecting ARC3 from Protease‐Mediated Degradation

**FIGURE 6:**
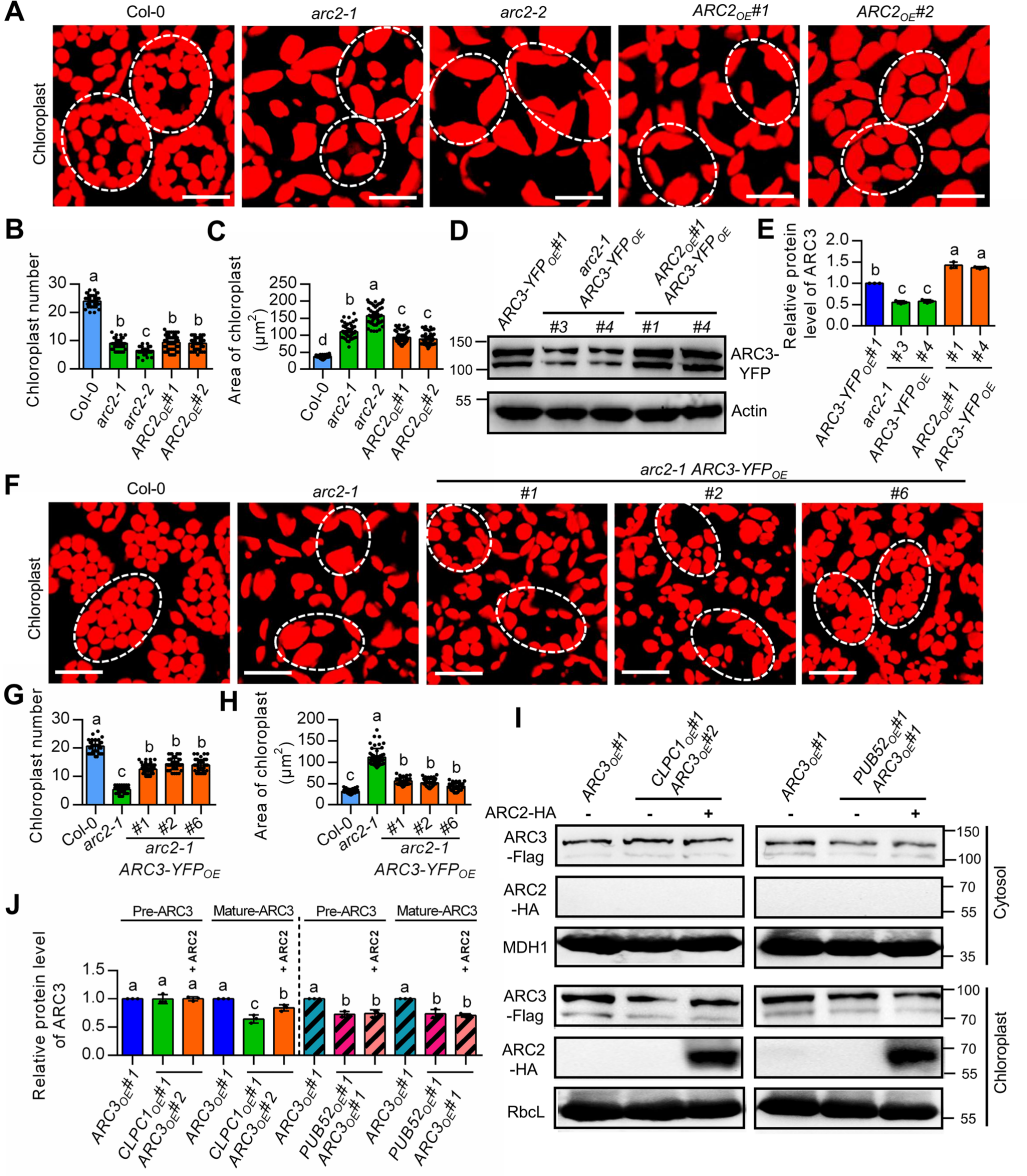
ARC2 positively regulates the chloroplast by stabilizing the ARC3 protein. (A) Chloroplast phenotypes in 4‐week‐old Col‐0, *ARC2‐RNAi* mutants (*arc2‐1* and *arc2‐2*) and *ARC2‐Flag* overexpression plants (*ARC2_OE_#1* and *ARC2_OE_#2*) through confocal microscopy. Dotted circles indicate mesophyll cells. Scale bar = 20 µm. (B, C) Quantitative analysis of chloroplast numbers per cell in a plane (B) and chloroplast area (C) of the indicated genotypes. Data presented are means ± SEM (three biological replicates, each with 50 cells per genotype). (D) ARC3‐YFP protein levels in 4‐week‐old *ARC3‐YFP_OE_#1*, *35S:ARC3‐YFP* stably expressing in *arc2‐1* and *ARC2_OE_#1* (*arc2‐1 ARC3‐YFP_OE_*, *ARC2_OE_#1 ARC3‐YFP_OE_*) lines with comparable *ARC3‐YFP* transcript levels. Plant actin was used as a loading control for total protein extractions. (E) Quantitative analysis of relative ARC3 protein level in chloroplast and cytosol of the indicated genotypes in (D). Data presented were means ± SEM (three biological replicates). Different letters above error bars indicate a significant difference at *p* < 0.05, using one‐way ANOVA with Tukey's test. (F) Chloroplast phenotypes of 4‐week‐old Col‐0, *arc2‐1*, and *arc2‐1 ARC3‐YFP_OE_* through confocal microscopy. Scale bar = 20 µm. (G, H) Quantitative analysis of chloroplast numbers per cell in a plane (G) and chloroplast area (H) of the indicated genotypes in (F). Data presented are means ± SEM (three biological replicates, each with 50 cells per genotype). Different letters above error bars indicate a significant difference at *p* < 0.05, using one‐way ANOVA with Tukey's test. (I) ARC3 levels in the chloroplast and cytoplasmic fractions of protoplasts from *ARC3_OE_#1*, *CLPC1_OE_#1 ARC3_OE_#2 and PUB52_OE_#1 ARC3_OE_#1* transgenic lines, with or without *35S:ARC2‐HA* transient expression. RbcL (large subunit of Rubisco) and MDH1 (Malate dehydrogenase) were used for chloroplast and cytoplasmic fraction purification control. (J) Quantitative analysis of the relative level of precursor and mature ARC3 in the chloroplast and cytosol of the indicated genotypes in (I). Data presented are means ± SEM (three biological replicates). Different letters above error bars indicate a significant difference at *p* < 0.05, using one‐way ANOVA with Tukey's test. The original blot can be found in Figure S11.

Although ARC2 is known to be involved in chloroplast division, its precise molecular mechanisms remain unclear. Due to the embryo lethality of homozygous *ARC2* T‐DNA insertion mutants [44], we generated *ARC2*‐silenced (*arc2*) plants using RNAi technology and *ARC2‐Flag* overexpression (*ARC2_OE_*) lines (Figure S7A). Both *arc2* and *ARC2_OE_* independent lines exhibited abnormal chloroplast division phenotypes, with fewer and larger chloroplasts per cell (Figure 6A–C), consistent with previous studies [44, 45]. qRT‐PCR analysis confirmed that *ARC2* does not influence *ARC3* mRNA levels (Figure S7B). Transient expression assays in *N. Benthamiana* epidermal cells indicated that ARC2 enhances ARC3 stability (Figure 2C). To validate this in *Arabidopsis*, we analyzed ARC3 protein levels in protoplasts from *arc2* and *ARC2_OE_* plants by transiently expressing *ARC3‐YFP*. When *ARC3‐YFP* expression levels were comparable across these genotypes (Figure S7C), ARC3 protein levels were significantly higher in *ARC2_OE_* and reduced in *arc2* compared to Col‐0 (Figure S7D), supporting a protective role of ARC2 against ARC3 degradation.

To further confirm that ARC2 protects the protein stability of ARC3 in plants, we generated stable transgenic plants expressing *35S:ARC3‐YFP* in *arc2‐1* and *ARC2_OE_#1* plants (Figure S7E). Consistent with protoplast results (Figure S7D), when *ARC3* expression levels were comparable, *ARC2_OE_#1 ARC3‐YFP_OE_* lines exhibited higher protein levels of ARC3, while *arc2‐1 ARC3‐YFP_OE_* lines showed reduced protein abundance compared to *ARC3‐YFP_OE_#1* (Figure 6D,E). To determine whether the abnormal chloroplast division phenotype in *arc2* mutants was caused by the reduced level of ARC3 protein, we observed the chloroplasts in *arc2‐1 ARC3‐YFP_OE_* lines. Expression of *ARC3‐YFP* in *arc2‐1* plants increased chloroplast number and reduced chloroplast size (Figure 6F–H; Figure S7E). These findings collectively demonstrate that ARC2 regulates chloroplast division by preventing excessive ARC3 degradation.

To identify the degradation pathway modulated by ARC2, we first examined whether co‐expression of *ARC2* could inhibit ARC3 degradation mediated by PUB52 or CLPC1 in *N. benthamiana* leaves. ARC2 partially suppressed CLPC1‐mediated degradation of ARC3‐LUC, but not on PUB52‐mediated degradation (Figure S8A–D). We then transiently transformed *ARC2‐HA* into protoplasts from *ARC3_OE_#1*, *CLPC1_OE_#1 ARC3_OE_#2*, and *PUB52_OE_#1 ARC3_OE_#1* leaves. Protein analysis revealed that *ARC2* specifically inhibited CLPC1‐mediated ARC3 degradation in chloroplasts, without affecting PUB52‐mediated cytosolic degradation (Figure 6I,J). These results establish that ARC2 specifically protects ARC3 from chloroplast protease‐mediated degradation.

During leaf development, ARC3 protein abundance declines as leaves expand [24]. To explore the mechanism underlying the decline in ARC3 protein abundance during leaf expansion, we analyzed the expression levels of *PUB52*, *CLPC1*, and *ARC2* in young versus expanding leaves. Quantitative analysis revealed a marked increase in *CLPC1* expression and a moderate decrease in *ARC2* expression in expanding leaves, whereas *PUB52* transcript levels remained comparable between the two developmental stages (Figure S8E). We then examined the corresponding protein levels in transgenic plants. As expected, ARC3 protein levels were markedly reduced in expanding leaves, consistent with a previous report [24]. Similarly, CLPC1 protein abundance was significantly higher in expanding leaves than in young leaves, while PUB52 protein levels showed no difference between the two stages (Figure S8F). These results collectively suggest that CLPC1 exerts a predominant role in modulating the ARC3 stability during leaf expansion.

## 3 Discussion

Chloroplast division is a complex process requiring coordinated action of multiple proteins and pathways to maintain organelle homeostasis. During leaf expansion, ARC3 protein levels in expanding leaves are markedly lower than those in young leaves, suggesting that ARC3 protein abundance is strictly controlled throughout plant development [24]. Our study reveals a sophisticated regulatory network centered on ARC3, a key inhibitor of FtsZ ring assembly [11], whose levels are modulated by both proteasomal and chloroplast proteolytic systems. We demonstrate that ARC3 abundance is precisely controlled through opposing mechanisms: degradation promoted by the E3 ubiquitin ligase PUB52‐mediated proteasome and the chloroplast protease chaperone CLPC1‐mediated proteolysis, and stabilization mediated by ARC2, collectively fine‐tuning chloroplast division.

Previous studies have proposed that proper ARC3 levels are crucial for normal chloroplast division [23, 42]. Our data further support this notion, showing a clear correlation between the severity of chloroplast division defects and ARC3 abundance (Figure 1A–D). We show that ARC3 is degraded through both the proteasome and chloroplast protease systems; inhibiting either pathway stabilizes ARC3 (Figure 1E–H). Three regulators─PUB52 (cytoplasmic/nuclear), CLPC1 (chloroplastic), and ARC2 (chloroplastic)─coordinately control ARC3 levels at distinct steps (Figure S3C). PUB52 ubiquitinates cytosolic ARC3 preproteins, targeting them for proteasomal degradation (Figure 4B–G); CLPC1 processes mature ARC3 in chloroplasts (Figure 5A,B); and ARC2 protects ARC3 from degradation (Figure 6D,E). Thus, ARC3 faces a two‐tiered, compartmentalized regulation: PUB52 acts as a cytosolic “first checkpoint” to limit precursor import into chloroplasts, while ARC2 and CLPC1 antagonistically fine‐tune mature ARC3 levels inside chloroplasts. This precise control ensures proper FtsZ ring assembly and chloroplast division. Although UPS components (such as CHIP and SP1) and chloroplast proteases have been implicated in protein quality control [32, 34], their specific roles in regulating division machinery components remained unclear. Our work bridges this gap by elucidating how these systems converge on ARC3 to modulate its stability.

Phenotypic analyses of *PUB52* and *CLPC1* mutants revealed chloroplast abnormalities, including reduced numbers, altered sizes (Figure 3A–F), and disrupted FtsZ ring assembly (Figure 3G), mirroring phenotypes observed with *ARC3* dysregulation. Notably, *pub52* mutants exhibited fewer, enlarged chloroplasts and discrete dots or short filaments of FtsZ2 (Figure 3A–C,G). In contrast, *CLPC1*‐*OE* plants had fewer chloroplasts but more FtsZ rings (Figure 3D–G). Biochemical assays confirmed that PUB52 promotes ARC3 degradation via ubiquitination in the cytoplasm (Figure 4A–C), while CLPC1 likely facilitates its proteolytic processing within chloroplasts. Genetic interaction analyses positioned both PUB52 and CLPC1 upstream of ARC3 in the regulatory hierarchy (Figure 4H–J and 5C–G; Figure S5E–G and S6C,D). These findings extend previous reports on UPS and protease involvement in chloroplast homeostasis by demonstrating their direct regulation of division machinery components [46].

Intriguingly, *clpc1‐c* mutants exhibited smaller and fewer chloroplasts (Figure 3D–F), contrasting with the enlarged chloroplasts in *ARC3* overexpressors (Figure 1B–D). This discrepancy may reflect the broader role of CLPC1 in chloroplast proteostasis, as its mutation causes significant disruption in protein homeostasis, resulting in stunted growth and chaperone accumulation [47]. Such proteostatic imbalance also compromises the transient transformation efficiency of the *FtsZ‐CFP* construct in protoplasts, resulting in weak fluorescence signals and consequently impeding reliable visualization of Z‐ring assembly in normally dividing chloroplasts (Figure 3G).

ARC2 (CPN60A), a plant homolog of bacterial GroEL, is known to stabilize proteins like TRXL1 [48]. Previous work showed that reduced ptCpn60α levels in *Arabidopsis* result in excessively stable FtsZ filaments [43]. Our study reveals that ARC2 specifically stabilizes ARC3 by counteracting CLPC1‐mediated degradation, without affecting PUB52‐dependent turnover. This compartment‐specific protection prevents excessive proteolytic cleavage of ARC3 in chloroplasts. Consistent with this model, ARC3 levels increased in *ARC2_OE_* plants and decreased in *arc2* mutants (Figure 6D,E). Transient assays in both *N. benthamiana* leaves and Arabidopsis protoplasts confirmed that ARC2 specifically inhibits CLPC1‐mediated ARC3 degradation but does not affect PUB52‐mediated degradation (Figure 6I,J; Figure S8A–D). Both *ARC2* knockdown and overexpression plants produced fewer and larger chloroplasts (Figure 6A–C), phenocopying *ARC3* mutation or overexpression lines. Importantly, *ARC3* overexpression effectively rescued the chloroplast division defects in *arc2* mutants (Figure 6F–H), supporting the model that multiple FtsZ rings in *arc2* mutants primarily result from reduced ARC3 levels [43]. Collectively, these findings establish ARC2 as a crucial stabilizer of ARC3 in chloroplast division regulation. Notably, unlike previous fixed‐cell methods, we assessed chloroplast morphology and number in living leaves by imaging the adaxial epidermis via confocal microscopy. We acquired over 50 images via 3D z‑stack scanning and merged them into a single composite. As this method might not capture all mesophyll chloroplasts, the resulting count was lower than that from fixed‐cell methods.

**FIGURE 7:**
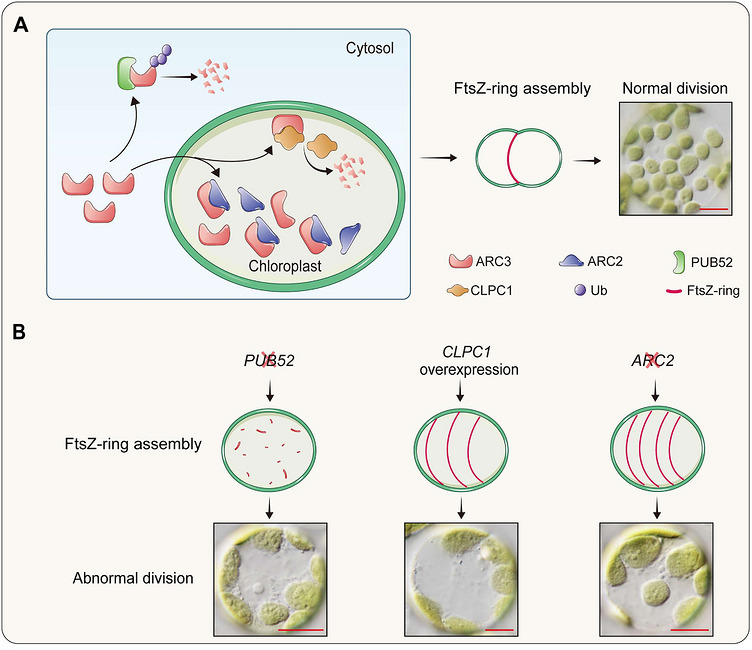
A proposed model for regulation of ARC3 stability during chloroplast division. The precise modulation of ARC3 protein levels is critical for ensuring proper chloroplast division. (A) In Col‐0, newly synthesized ARC3 is translocated into chloroplasts from cytoplasm, where PUB52‐mediated ubiquitination targets excess ARC3 for proteasomal degradation. Within chloroplasts, ARC3 stability is regulated by a balance between opposing actions of ARC2 stabilization and CLPC1‐dependent proteolysis, maintaining optimal levels for the assembly of the FtsZ ring at the mid‐plane chloroplast division site and normal chloroplast division. (B) Disruptions to this equilibrium, whether through *ARC2* depletion, *CLPC1* overexpression, or *PUB52* mutation, lead to ARC3 accumulation or depletion, resulting in defective FtsZ‐ring formation and aberrant chloroplast division. Scale bars, 10 µm. These findings reveal an exquisite dual‐compartment regulatory network that coordinates cytoplasmic and chloroplast proteostasis to ensure faithful organelle division, highlighting ARC3 as a central role in chloroplast division control.

Our work highlights the stringent, multi‐layered control of ARC3 protein levels (Figure 7). Newly synthesized ARC3 in the cytoplasm is first monitored by the ubiquitin‐proteasome system before chloroplast import. Following translocation, it undergoes additional quality control by the CLP protease system during chloroplast division regulation. To prevent excessive degradation, ARC2 stabilizes ARC3 by antagonizing CLPC1, maintaining optimal ARC3 levels for proper division control.

Several questions remain for future investigation: (1) The precise mechanism of CLPC1‐mediated ARC3 degradation requires clarification─whether CLPC1 directly cleaves ARC3 or recruits other proteases; (2) The abundance of the reduced form of ARC3 is high in young leaves but decreases sharply with leaf aging, indicating that the reduced form of ARC3 protein plays a more critical role in regulating chloroplast division. The physiological relevance of ARC3 redox regulation, suggested by the rescue of *arc3‐2* phenotypes through cysteine mutation, requires further exploration; (3) The spatial and temporal dynamics of ARC3 degradation during cell cycle progression or under stress conditions remain to be elucidated.

In conclusion, our study uncovers a multi‐layered regulatory network controlling ARC3 stability, integrating both cytoplasmic and chloroplast proteostasis systems to ensure proper chloroplast division. By identifying PUB52, CLPC1, and ARC2 as key regulators, we provide a conceptual framework for understanding how post‐translational modifications fine‐tune organelle dynamics.

## 4 Methods

### 4.1 Plant Materials and Growth Conditions

The *Arabidopsis thaliana* Columbia (Col‐0) ecotype was the genetic background for all transgenic lines utilized in this study. The *arc3‐2* mutant (SALK_057144) was obtained from ABRC stock. The *arc3RNAi* and *arc2* mutants were generated via RNA interference (RNAi) technology [49]. Single mutants *pub52‐c* and *clpc1‐c* were created using CRISPR/Cas9‐mediated genome editing [42]. Double mutants *pub52arc3* and *clpc1arc3* were constructed through genetic crossing of *arc3‐2* with *pub52‐c1* or *clpc1‐c1*. Plants were cultivated under controlled conditions at 21°C, with a photoperiod of 16 h light / 8 h darkness, light intensity of 70 µmol m^−^^2^ s^−^^1^, and relative humidity of 60%.

### 4.2 Plasmid Construction and Generation of Transgenic Plants

The coding sequences (CDS) (without stop codons) of target genes were amplified from *Arabidopsis* cDNA and fused to YFP in the pGreen0179‐35S‐GWB‐YFP vector [50], resulting in constructs of ARC3‐YFP, PUB52‐YFP, CLPC1‐YFP, ARC2‐YFP, and FtsZ2‐YFP. Additionally, *ARC3* and *ARC2* CDS without stop codons were amplified and fused to a Flag‐tag at the C‐terminus in the pCambia2306 vector. To construct ARC3pro:ARC3‐YFP, a 1604‐bp genomic fragment upstream of the start codon of *ARC3* was amplified using primers listed in Table S1. This fragment was cloned into the pGreen0179‐35S‐GWB‐YFP vector via *Kpn*I and *Hind*III sites. The full‐length CDS of *ARC3* was then amplified and integrated into the modified pGreen0179‐ARC3pro‐GWB‐YFP vector through a Gateway cloning strategy, resulting in the final construct as ARC3pro:ARC3‐YFP [51].

The respective constructs were introduced into Agrobacterium tumefaciens strain GV3101 and subsequently transformed into plants using the floral dip method [52].

### 4.3 Chloroplast Phenotype Analysis

Expanded rosette leaves from 3‐ to 4‐week‐old plants were harvested for the analysis of chloroplast phenotypes. Chloroplasts in mesophyll cells were visualized using a Leica TCS SP8 confocal microscope equipped with a 63× water immersion objective. Using confocal microscopy, we acquired over 50 images through 3D z‑stack scanning with the z‑axis focus range set to 22–25 µm. These images were merged into a single composite image. Images of chloroplasts captured under the microscope were analyzed for both chloroplast number and size using ImageJ software (http://rsb.info.nih.gov/ij/).

For chloroplast number quantification, chloroplasts in 50 mesophyll cells per genotype were counted at a single focal plane per cell. The average number of chloroplasts per cell was then calculated. For chloroplast area measurement, the polygon tool in ImageJ was used to trace individual chloroplast boundaries, and the pixel area within these regions was calculated by the software. This area was converted to actual dimensions using a scale bar reference (1 mm^2^ pixel = 0.00011 µm^2^). The average chloroplast area was determined by analyzing 50 chloroplasts per genotype.

### 4.4 Semi‐Quantitative PCR and Quantitative Real‐Time PCR

Total RNA was isolated from expanded rosette leaves or protoplasts of 3‐ to 4‐week‐old plants utilizing TRIzol reagent (Invitrogen Life Technologies, Carlsbad, CA, USA), following the manufacturer's protocol. Complementary DNA (cDNA) synthesis was carried out via reverse transcription using M‐MLV reverse transcriptase (Promega, Madison, WI, USA). *Actin7* served as the internal reference for semi‐quantitative RT‐PCR amplification. For quantitative analysis, real‐time PCR was conducted using a SYBR Green Master Mix (ABclonal, Wuhan, China) on a C1000 Touch Thermal Cycler detection system (Bio‐Rad, Hercules, CA, USA), with the *EF1α* gene used as the internal control. All primer sequences are detailed in Table S1.

### 4.5 Immunoprecipitation‐Mass Spectrometry (IP‐MS) Analysis

For IP‐MS experiments, leaf tissues (10 g) from 4‐week‐old transgenic plants were immersed in liquid nitrogen and homogenized in 30 mL of ice‐cold lysis buffer (50 mm Tris‐HCl, pH 7.5, 150 mm NaCl, 0.5 mm EDTA, 0.5% Nonidet P‐40, 1× protease inhibitor cocktail). Lysates were obtained by centrifugation at 12 000 × *g* for 15 min at 4°C. Supernatants underwent immunoprecipitation with 100 µL of GFP‐Trap magnetic agarose beads (Smart‐Lifesciences, Changzhou, China, SM038005) under gentle agitation for 4 h at 4°C. Immunocomplexes were washed five times with stringent wash buffer (50 mm Tris‐HCl pH 7.5, 150 mm NaCl, 0.5 mm EDTA) to remove nonspecific interactions. Immunoprecipitated proteins were eluted through thermal denaturation in Laemmli buffer at 95°C for 10 min before being resolved via 10% SDS‐PAGE, and visualized using Coomassie Brilliant Blue R‐250 staining. Gel‐excised protein bands underwent in‐gel tryptic digestion followed by peptide desalting and enrichment prior to LC‐MS/MS analysis.

### 4.6 Bimolecular Fluorescence Complementation (BiFC) and Split‐Luciferase (Split‐LUC) Assays

For BiFC analysis, CDS of *ARC3*, *CLPC1*, *PUB52*, and *ARC2* (excluding stop codons) were amplified and cloned into either the nYFP‐tagged pEarleygateYN or cYFP‐tagged pEarleygateYC binary vectors via Gateway recombination (Thermo Fisher Scientific). For split‐LUC assays, full‐length CDS of *ARC3*, *PUB52*, *CLPC1*, *ARC2*, *CCT3*, *RD21A*, *FTSH2*, *CLPR2*, and *SBT1.8* (with or without stop codons) were amplified (primers listed in Table S1) and subcloned into the C‐terminal luciferase (JW772‐cLUC) or N‐terminal luciferase (JW771‐nLUC) vectors [53]. All constructs were transformed into *Agrobacterium tumefaciens* strain GV3101 and co‐infiltrated with P19 into *N. benthamiana* leaves with a combination of an OD_600_ of 0.6, or 20 µg plasmids were transformed into mesophyll protoplasts isolated from 4‐week‐old *Arabidopsis* Col‐0 plants via polyethylene glycol‐mediated transformation [54]. Infiltrated *N. benthamiana* plants were grown at controlled conditions for 2 days, and transfected protoplasts were incubated under low‐light conditions (21°C for 16 h) prior to imaging.

For BiFC analyses, reconstituted YFP fluorescence was visualized using a Leica SP8 confocal laser scanning microscope with excitation at 514 nm and emission between 525 nm and 550 nm. For Split‐LUC assays, after 48 h, 1 mm luciferin (LUCK‐1G; GOLDBIO, Missouri, USA) was applied uniformly to the surface of the leaves, and the images were captured in the dark using a Lumazone CA low‐light CCD imaging system.

### 4.7 Pull‐Down Assay

Recombinant proteins GST‐ARC3, GST (negative control), MBP‐PUB52, His‐CLPC1N, and His‐ARC2 were expressed in *E. coli* BL21(DE3) under optimized conditions (0.5 mm IPTG, 16°C for 20 h). Affinity purification was performed using tag‐specific resins: Glutathione Agarose (Smart‐Lifesciences, Changzhou, China, SA010025) for GST‐ARC3 and GST, Dextrin Beads 6FF (Smart‐Lifesciences, SA026025) for MBP‐PUB52, and Ni IDA Beads 6FF (Smart‐Lifesciences, SA052025) for His‐CLPC1N and His‐ARC2. After purification, equimolar concentrations of GST or GST‐ARC3 were co‐incubated with MBP‐PUB52, His‐CLPC1N, and His‐ARC2 in PBS buffer (pH 7.4) at 4°C for 4 h with gentle agitation. The beads were then washed five times with washing buffer (20 mm Tris‐HCl, pH 7.5, 100 mm NaCl, 1 mM EDTA, 0.5% Nonidet P‐40). Proteins were eluted from beads by boiling in 100 µL 2×SDS sample buffer and separated on a 10% SDS‐PAGE gel. Gel blots were detected using anti‐His (ABclonal, Wuhan, China, AE003, 1:5000 dilution), anti‐GST (ABclonal, AE001, 1:5000 dilution), and anti‐MBP (ABclonal, AE016, 1:5000 dilution) antibodies.

### 4.8 Co‐Immunoprecipitation (Co‐IP) Assays

The *35S*:*ARC2‐Flag* construct was transformed into *35S:ARC3‐YFP* and *35S:YFP* plants, and *35S*:*ARC3‐Flag* was introduced into *35S:PUB52‐YFP*, *35S:CLPC1‐YFP*, or *35S:YFP* transgenic plants through floral dip transformation. Proteins from leaf tissue from 4‐week‐old transgenic plants were isolated. Immunoprecipitation was conducted by incubating 2 mg of input protein with 25 µL of GFP‐Trap magnetic agarose beads (Smart‐Lifesciences, Changzhou, China, SM038005) for 4 h on a rotator at 4°C. The bead‐bound complexes underwent three washes with wash buffer (50 mM Tris‐HCl pH 7.5, 150 mm NaCl, 0.5 mm EDTA), followed by elution in 2× Laemmli buffer at 95°C for 5 min. The eluates were resolved via SDS‐PAGE and transferred to PVDF membranes. Membranes were blocked with non‐fat dry milk in TBST [20 mm Tris‐HCl pH 7.6, 150 mm NaCl, 0.1% (v/v) Tween‐20] for 2 h at room temperature before probing overnight at 4°C with monoclonal anti‐GFP (1:2,000, ABclonal, Wuhan, China, AE012) or anti‐Flag (1:1,000, ABclonal, AE005) primary antibodies. After three washes with TBST, membranes were incubated with HRP‐conjugated anti‐mouse IgG secondary antibody (1:10,000, ABclonal, AS003) for 1 h at 25°C. Protein interactions were visualized using Pierce ECL Western Blotting Substrate (LANTOBIO, Wuhan, China) with chemiluminescent detection on a Bio‐Rad ChemiDoc MP system.

### 4.9 In Vitro Ubiquitination Assay

Ubiquitination assays were performed with modifications as described [55]. Recombinant GST‐ARC3 and MBP‐PUB52 were expressed in *E.coli* BL21 (DE3) under optimized conditions (0.5 mm IPTG, 16°C, 20 h) and purified via affinity chromatography using Glutathione Agarose (Smart‐Lifesciences, Changzhou, China SA010025) or Dextrin Beads 6FF (Smart‐Lifesciences, SA026025), respectively. Reaction mixtures containing 300 ng of GST‐ARC3 and equimolar amounts of MBP‐PUB52 were incubated with 50 ng of ubiquitin‐activating enzyme E1 (Beyotime, Shanghai, China, #P1001), 100 ng of ubiquitin‐conjugating enzyme E2 (Beyotime, #P1002), and 5 µg of ubiquitin (Beyotime, #P1003) or not in a reaction buffer (50 mm Tris‐HCl pH 7.4, 50 mm HEPES‐KOH, 5 mm MgCl_2_, 2 mm ATP, 2 mM DTT). Enzymatic reactions were carried out at 30°C for 2 h with gentle agitation. Ubiquitination levels were determined by Western blotting using a rabbit polyclonal anti‐ubiquitin antibody (1:1,000; Cell Signaling Technology, Boston, USA, #3936).

### 4.10 Semi‐In Vitro Ubiquitination Assay

Recombinant GST‐ARC3 was immobilized by Glutathione Agarose beads (Smart‐Lifesciences, SA010025). Total proteins were extracted from *Arabidopsis* leaves using ice‐cold extraction buffer [25 mm Tris‐HCl pH 7.5, 10 mm NaCl, 10 mm MgCl_2_, 4 mm PMSF (freshly added), 5 mm DTT, 10 mm ATP]. Immobilized GST‐ARC3 complexes were incubated with clarified lysates from Col‐0, *pub52‐c*, or *PUB52_OE_* plants in reaction buffer [25 mM Tris‐HCl pH 7.5, 150 mm NaCl, 0.5% (v/v) Triton X‐100, 1 mM PMSF, 2 mm DTT, 100 µm MG132] at 22°C for 3 h with rotation. Beads‐bound proteins were washed with increasing stringency buffer [25 mm Tris‐HCl pH 7.5, 300–600 mM NaCl, 0.5% (v/v) Triton X‐100, 1 mM PMSF, 2 mM DTT], and eluted by thermal denaturation in 1× Laemmli buffer (95°C, 10 min) and resolved by SDS‐PAGE. Ubiquitination and protein levels of ARC3 were determined by Western blotting using a rabbit polyclonal anti‐ubiquitin antibody (1:1,000; Cell Signaling Technology, Boston, USA, #3936) and anti‐GST (1:2,000; Abcam, Cambridge, UK, #ab9085). Actin was used as a loading control (anti‐Actin, 1:5,000; Agrisera, Shanghai, China, #AS13 2640).

### 4.11 Cell‐Free Protein Degradation Assay

Total proteins were extracted from 4‐week‐old Arabidopsis Col‐0, *pub52‐c*, and *PUB52_OE_* plants using ice‐cold extraction buffer (50 mm Tris‐HCl pH 7.5, 10 mm NaCl, 10 mm MgCl_2_, 5 mm DTT, 1× protease inhibitor cocktail), and quantified via Bradford assay. Purified recombinant GST‐ARC3 protein (200 ng) was incubated with 50 µg of plant protein extracts in degradation buffer (50 mm Tris‐HCl pH 7.5, 10 mm MgCl_2_, 5 mm ATP, 1 mm DTT) at 22°C for 0–60 min with or without 100 µm of MG132 (MedChemExpress, New Jersey, USA, HY‐13259). Aliquots were collected at 0 min, 15 min, 30 min, and 60 min, and terminated by addition of 2× Laemmli buffer, followed by denaturation at 95°C for 5 min. Degradation kinetics were assessed by immunoblotting using monoclonal anti‐GST antibody (1:2,000; Abcam, Cambridge, UK, #ab9085), with anti‐Actin (1:5,000; Agrisera, Shanghai, China, #AS13 2640) as a loading control. Band intensities were quantified using Image Lab software (Bio‐Rad).

### 4.12 Isolation of Cytosolic and Chloroplast Proteins from Protoplasts

After overnight transformation, protoplasts from four independent reactions were pooled, pelleted, and resuspended in 100 µL of GR buffer (50 mm HEPES, 0.33 M sorbitol, 10 mm EDTA, and 0.5 mg/mL BSA). Samples were kept on ice for 20 min, then lysed by alternating vortexing (20 s at max speed) and ice‐chilling (30 s), supplemented with repeated pipetting using a narrow‐tip (10‐µL tip fitted onto a 1‐mL tip). Lysis progress was monitored microscopically (40× objective) until complete membrane disruption and chloroplast dispersion were observed. The lysate was centrifuged at 6,000 × *g* for 10 min at 4°C. The supernatant (cytosolic fraction) was collected, transferred to a new 1.5‐mL tube, mixed with an equal volume of protein extraction buffer, and kept on ice. The pellet was resuspended in 500 µL of GR buffer (50 mm HEPES, 0.33 m sorbitol) and centrifuged at 1,000 × *g* for 10 min at 4°C to remove residual nuclei; the supernatant was collected. This supernatant was centrifuged again at 6,000 × *g* for 10 min at 4°C. The wash was repeated several times for thorough purification. After the final wash, the pellet was resuspended in 200 µl of protein extraction buffer to yield the chloroplast fraction. Fraction Purity of both fractions was confirmed by Western blotting using antibodies against MDH1 (ABclonal, Wuhan, China, A7563, 1:2000 dilution) and RbcL (ABclonal, A23203, 1:2000 dilution).

### 4.13 Statistical Analysis

The study performed all quantitative analyses and real‐time PCR experiments with a minimum of three biological replicates, and data are presented as the mean ± standard error of the mean (SEM). Protein abundance and LUC intensity were normalized to the control group (control mean = 1). For data visualization, *p*‐values were determined using the unpaired Student's *t*‐test or one‐way ANOVA, as appropriate. GraphPad Prism 9 was employed to generate bar graphs and heatmaps. Statistical evaluations were conducted in GraphPad Prism 9, employing ANOVA Tukey's test, with consideration given to statistical normality and homogeneity of variance. In the figures, distinct letters marked above error bars indicate statistically significant differences (*p* < 0.05).

## Author Contributions

H.H. conceived and designed the research. Y.Y., D.X., and R.L. performed most of the experiments and contributed equally. Y.Y., D.X., R. L., Z.Y., and Z.W. analyzed the data. Y.Y., D.X., R.L., and H.H. wrote the manuscript. Other authors assisted in the experiments and discussed the results. All authors read and approved the manuscript.

## Conflicts of Interest

The authors declare no conflict of interest.

## Supporting information




**Supporting File**: advs75715‐sup‐0001‐SuppMat.docx.

## Data Availability

The data that support the findings of this study are available from the corresponding author upon reasonable request.
