# Supplementary material for: Dual Cytoplasmic and Chloroplastic Mechanisms Fine‐Tune Chloroplast Division through ARC3 Protein Stability
Source: Adv Sci (Weinh). 2026 May 26;13(45):e23660. doi: 10.1002/advs.202523660 (PMC13335998; doi:10.1002/advs.202523660)
Supplement: Supplementary file 1 [file ADVS-13-e23660-s001.docx]

Supporting Information

**Dual Cytoplasmic and Chloroplastic Mechanisms Fine-Tune Chloroplast Division through ARC3 Protein Stability**

Yang Yuan, Denghao Xiang, Ruiying Li, Zhichuang Yue, Zhipeng Wang and Honghong Hu^*^

**Figure S1.** Reduced status of ARC3 protein plays an important role during chloroplast division. (A) The smaller band is the oxidized state of the ARC3 protein. Recombinant GST-ARC3, GST-ARC3^9C-S^ (ARC3^9C-S^, Cys 42, 51, 54, 65, 93, 207, 299, 314, 611 mutated into Ser), GST-ARC3^10C-S^ (all ten Cys mutated into Ser) and GST-ARC3^∆TP^ (deletion of transit peptide) in the pGEX-4T-1 vector were expressed in *E. coli* strain BL21 (DE3) and detected using anti-GST antibody. Red represents reduced status and ox represents oxidized status of GST-ARC3. (B) The oxidized and reduced states of the ARC3 protein level in 4-, 5-, 6-week-old transgenic *ARC3-YFP_OE_*_,_ plant respectively. Plant actin was used as loading control for total protein extractions. The ratio of the oxidized form to the reduced form of ARC3-YFP was quantified by ImageJ and marked below the bands. (C)The chloroplast phenotypes (top panels) and ARC3-YFP fluorescence (bottom panels) in 4-week-old Col-0, *arc3-2*, and *arc3-2* complementation lines stably expressing *ARC3_pro_:ARC3-YFP* or *ARC3_pro_:ARC3^9c-s^-YFP* through confocal microscopy. Scale bar = 10 μm. Original blot can be found in Figure S10.

**Figure S2.** The influence of putative interactors of ARC3 on its luciferase intensity. (A) Classiﬁcation of ARC3 interactors identified by IP-MS into biological processes. X axis represents biological processes of DAVID enrichment analysis. Y axis represents numbers of identified proteins. (B-I) The relative LUC activity of ARC3-LUC co-expressing with its interactors or not in Figure 2C. The LUC intensity was normalized using averaged expression levels of *LUC*. Data are means ± SEM (three biological replicates). Different letters above error bars indicated significant difference at *p* < 0.05, using one-way ANOVA with Tukey’s test.

**Figure S3.** The subcellular localization of ARC3, PUB52, ARC2, and CLPC1. (A) Localization of YFP (upper panels), ARC3-YFP (middle panels) in protoplasts of Col-0 and ARC3-YFP in the transgenic plants expressing *35S:ARC3-YFP* (bottom panels). The white arrows indicate the cytoplasmic localization of ARC3-YFP. BF, bright field. Scale bar = 10 μm. (B) ARC3-YFP protein levels in chloroplastic and cytoplasmic fractions from *ARC3-YFP_OE_#3* plants by western blot. RbcL (large subunit of Rubisco) and MDH1 (Malate dehydrogenase) were used for chloroplast and cytoplasmic fraction purification control. (C) Localization of PUB52-YFP, ARC2-YFP, and CLPC1-YFP in their corresponding stable transgenic plants, respectively. Scale bar = 10 μm. (D) Coimmunoprecipitation (Co-IP) assays of ARC3 and PUB52 in cytoplasm and chloroplasts isolated from *PUB52_OE_#1 ARC3_OE_#1* transgenic plants. RbcL (large subunit of Rubisco) and MDH1 (Malate dehydrogenase) were used for chloroplast and cytoplasmic fraction purification control. Original blot can be found in Figure S10.

**Figure S4.** Identification of *PUB52* and *CLPC1* mutants by CRISPR-Cas9 technology. (A) Gene model of *PUB52* and its CRISPR targets and mutation sites in *pub52-c* mutants. (B) The PUB52-YFP protein levels in 4-week-old Col-0 and *PUB52_OE_* plants. Plant actin was used as loading control for total protein extractions. (C) Gene model of *Arabidopsis CLPC1* and its CRISPR targets and mutation sites in *clpc1-c* mutants. (D) The CLPC1-YFP protein levels in 4-week-old Col-0 and *CLPC1_OE_* plants. Plant actin was used as loading control for total protein extractions. (E) Expression levels of *CFP* in Col-0, *pub52-c1*, *pub52-c2*, *PUB52_OE_#1*, *PUB52_OE_#2, clpc1-c1*, *clpc1-c2* and *CLPC1_OE_#1, CLPC1_OE_#2* protoplasts transiently expressing *FtsZ2-CFP* by RT-PCR. *ACTIN7* was used as internal control. (F) Proportion of different assembly forms of FtsZ2 in each genotype in (E). Each genotype with 50 cells per experiment. Original blot and gel image can be found in Figure S10.

**Figure S5.** PUB52 regulates chloroplast division mainly through ARC3. (A) *ARC3* expression levels in 4-week-old Col-0, *pub52-c* mutants and *PUB52* overexpression plants (*PUB52_OE_*). (B) *ARC3* expression levels in 4-week-old Col-0, *pub52-c1* mutant and *PUB52_OE_#1* plants stably expressing *35S:ARC3-Flag* (*ARC3_OE_*, *pub52-c1 ARC3_OE_* and *PUB52_OE_#1 ARC3_OE_*). Expression levels in (A and B) were normalized to *EF1α*. Data are means ± SEM (three biological replicates). Different letters above error bars indicate significant difference at *P* < 0.05, using one-way ANOVA with Tukey’s test. ns, no significant difference. (C) Expression levels of *YFP* in Col-0, *pub52-c1*, *arc3-2*, *pub52arc3* protoplasts transiently expressing *FtsZ2-YFP* by RT-PCR. *ACTIN7* was used as internal control. (D) Proportion of different assembly forms of FtsZ2 in each genotype in (C). Each genotype with 50 cells per experiment. (E) The chloroplasts in 4-week-old Col-0, *PUB52_OE_#1,* *PUB52_OE_#1* plants stably expressing *35S:ARC3-Flag* leaves. Scale bar = 20 μm. Dotted circles indicate mesophyll cells. (F, G) Quantitative analysis of chloroplast numbers per cell in a plane (F) and chloroplast size (G) of the indicated genotypes in (E). Data are means ± SEM (three biological replicates, each with 50 cells per genotypes). Different letters above error bars indicate significant difference at *p* < 0.05, using one-way ANOVA with Tukey’s test. Original gel image can be found in Figure S11.

**Figure S6.** *CLPC1* participates in the chloroplast division process upstream of *ARC3*. (A) *ARC3* expression levels in 4-week-old Col-0, *clpc1-c* mutants and *CLPC1* overexpression plants (*CLPC1_OE_*). Expression levels were normalized to *EF1α*. Data are means ± SEM (three biological replicates). (B) *ARC3* expression levels in 4-week-old Col-0, *clpc1-c1* and *CLPC1_OE_#1* stably expressing *35S:ARC3-Flag* lines (*ARC3_OE_*, *clpc1-c1* *ARC3_OE_* and *CLPC1_OE_#1 ARC3_OE_*). Expression levels were normalized to *EF1α*. Data are means ± SEM (three biological replicates). (C) The mesophyll cell chloroplasts in the 4-week-old Col-0, *clpc1-c1*, *arc3-2*, and *clpc1arc3* double mutant through confocal microscopy. Dotted circles indicate mesophyll cells. Scale bar = 10 μm. (D) Quantitative analysis of chloroplast numbers in a plane of the indicated genotypes in (B). Data presented are means ± SEM (three biological replicates, each with 50 cells per genotypes). Different letters above error bars indicate significant difference at *p*< 0.05, using one-way ANOVA with Tukey’s test. (E) Expression levels of *CFP* in Col-0, *CLPC1_OE_#1*, and *CLPC1_OE_#1* stably expressing *35S:ARC3-Flag* lines (*CLPC1_OE_#1 ARC3_OE_*) protoplasts transiently expressing *FtsZ2-CFP* by RT-PCR. *ACTIN7* was used as internal control. Original gel image can be found in Figure S11.

**Figure S7.** ARC2 can stabilize the ARC3 protein. (A, B) Quantitative PCR analyses of *ARC2* (A) and *ARC3* (B) expression levels in 4-week-old Col-0, *arc2* and *ARC2_OE_* plants. Expression levels were normalized to *EF1α*. Data are means ± SEM (three biological replicates). (C) Expression levels of *YFP* in Col-0, *arc2*, and *ARC2_OE_* protoplasts transiently expressing *ARC3-YFP* by RT-PCR. *ACTIN7* was used as internal control. (D) ARC3-YFP protein levels in the protoplasts expressing *35S:ARC3-YFP* in (C). Plant actin was used as loading control for total protein extractions. Relative fold changes of ARC3-YFP to loading controls were quantified by Image J and marked below the band. (E) *ARC3* expression levels in 4-week-old Col-0, *arc2-1* and *ARC2_OE_#1* stably expressing *35S:ARC3-YFP* plants. Expression levels were normalized to *EF1α*. Data are means ± SEM (three biological replicates). Different letters above error bars indicate significant difference at *p* < 0.05, using one-way ANOVA with Tukey’s test. Original blot and gel image can be found in Figure S11.

**Figure S8.** The impact of ARC2 on PUB52- and CLPC1-mediated ARC3 degradation. (A, B) The effect of ARC2 on CLPC1-mediated ARC3-LUC degradation in *N. benthamiana* leaves (A) and its quantification (B). (C, D) The effect of ARC2 on PUB52-mediated ARC3-LUC degradation in *N. benthamiana* leaves (C) and its quantification (D). The LUC intensity was normalized using averaged expression levels of LUC and plotted as relative values. Data are means ± SEM (three biological replicates). Different letters above error bars indicated significant difference at *p* < 0.05, using one-way ANOVA with Tukey’s test. (E) *PUB52*, *CLPC1* and *ARC2* genes expression levels in young and expanding leaves of Col-0. Expression levels were normalized to *EF1α*. Data are means ± SEM (three biological replicates). (F) Detection of ARC3, PUB52, CLPC1 and ARC2 protein levels in young and expanding leaves of *ARC3-YFP_OE_*, *PUB52-YFP_OE_,* *CLPC1-YFP_OE_*, and *ARC2-YFP_OE_* plants. Plant actin was used as loading control for total protein extractions. Relative fold changes of the indicated protein to loading controls were quantified by Image J and marked below the band. Original blot can be found in Figure S11.

**Figure S9.** Replicates of Western-blots. (A) ARC3 protein levels in *ARC3-YFP* stably expressing plants (*ARC3-YFP_OE_#1*) treated with proteasome inhibitor MG132 (middle panel) or protease inhibitor cocktail (PIC) (bottom panel) or not (control; upper panel). (B) ARC3 protein levels in *ARC3-YFP* stably expressing plants (*ARC3-YFP_OE_#1*) treated with Cycloheximide (CHX) (middle panel) or CHX with MG132 (lower panel) or CHX with protease inhibitor cocktail (PIC) (bottom panel) or not (CK; upper panel). (C) The smaller band is the oxidized state of the ARC3 protein. (D) Semi-in vitro ubiquitination assays of GST-ARC3 proteins with protein extracts from 4-week-old Col-0, *pub52-c1* or *PUB52_OE_#1* plants. (E) Cell-free degradation assay of ARC3 degradation by PUB52. Purified GST-ARC3 was incubated with protein extracts from 4-week-old Col-0, *pub52-c1* or *PUB52_OE_#1* plants. (F) ARC3 protein (Pre-protein and Mature-protein) levels in 4-week-old Col-0, *pub52-c1* and *PUB52_OE_#1* plants stably expressing *35S:ARC3-Flag*. (G) ARC3 protein (Pre-protein and Mature-protein) levels in 4-week-old *ARC3_OE_#1*, *35S:ARC3-Flag* overexpressing *clpc1-c1* (*clpc1-c1 ARC3_OE_*) and *CLPC1_OE_#1* (*CLPC1_OE_#1 ARC3_OE_*) lines. (H) ARC3-YFP protein levels in 4-week-old *ARC3-YFP_OE_#1*, *35S:**ARC3-YFP* stably expressing in *arc2-1* and *ARC2_OE_#1* (*arc2-1 ARC3-YFP_OE_*, *ARC2_OE_#1 ARC3-YFP_OE_*) lines. (I) ARC3 levels in the chloroplast and cytoplasmic fractions of protoplasts from *ARC3_OE_#1*, *CLPC1_OE_#1 ARC3_OE_#2 and PUB52_OE_#1 ARC3_OE_#1* transgenic lines, with or without *35S:ARC2-HA* transient expression. Original blot can be found in Figure S12.

**Figure S10.** Original unprocessed blots corresponding to the main and supplementary figures. Original unprocessed blots for the Western blot panels shown in Figures 1E, 1G, S1A, S1B, 2E-J, S3B, S3D, S4B, S4D, and gel images for RT-PCR in Figure S4E, which respectively arranged in alphabetical order as shown in the figure.

**Figure S11.** Original unprocessed blots corresponding to the main and supplementary figures. Original unprocessed blots for the Western blot panels shown in Figures 4A-D, 4F, 5A, 6D, S7D, 6I, S8F, and gel images for RT-PCR in Figures S5C, S6E, S7C, which respectively arranged in alphabetical order as shown in the figure.

**Figure S12.** Original unprocessed blots corresponding to the main and supplementary figures. Original unprocessed blots for the Western blot panels shown in Figures S9A-I, which respectively arranged in alphabetical order as shown in the figure.

| **primer** | **usage** | **Sequence（5’-3’）** |
| --- | --- | --- |
| ARC3-RNAi-F | RNAi | TCCCCCGGGCATGCCATGGATGCCGATTTCTATGGAACTTC |
| ARC3-RNAi-R |  | CGGGATCCTTGGCGCGCCCAAAATCGCAGAGGAAGAGAA |
| ARC2-RNAi-F1 |  | CATTTCATTTGGAGAGGACAATGGCGTCTGCAAACGCTCTC |
| ARC2-RNAi-R1 |  | TAATGTAACATAAGAAATTCAATGGTGACTCCATCATTCAC |
| ARC2-RNAi-F2 |  | CACATCTTTTTTGGTCAATTATGGCGTCTGCAAACGCTCTC |
| ARC2-RNAi-R2 |  | TCTCATTAAAGCAGGACTCTAATGGTGACTCCATCATTCAC |
| PUB52-401E-F | CRISPR/Cas9 | ATATATGGTCTCGATTGCTGTCAAGGTCCTTCACGCGTTTTAGAGCTAGAAATAGC |
| PUB52-401E-R |  | ATTATTGGTCTCGAAACGATCGTACGTGTACCCATCCAATCTCTTAGTCGACTCTAC |
| CLPC1-401E-F |  | ATATATGGTCTCGATTGGGCCCAAGGGGAAAGCTAGGTTTTAGAGCTAGAAATAGC |
| CLPC1-401E-R |  | ATTATTGGTCTCGAAACTGCGTATCACCTAGCCATCCAATCTCTTAGTCGACTCTAC |
| ARC3-4T-1-F  ARC3-4T-1-R  PUB52-C2X  PUB52-C2X-R  CLPC1-32a-F  CLPC1-252-32a-R  ARC2-32a-F  ARC2-32a-R | Prokaryotic expression | CTGGTTCCGCGTGGATCCATGCCGATTTCTATGGAACTTC  GAGTCGACCCGGGAATTCATCTCCGGCGTCCACTTGTTTC  AGGGAAGGATTTCAGAATTCATGGAAGAGAAAAAAGTAGTAAGAG  AGGTCGACTCTAGAGGATCCCCGTTTATTAGATTTCCACTCCA  CCATGGCTGATATCGGATCCATGGCTATGGCCACAAGGGTG  CAAGCTTGTCGACGGAGCTCATTGCTGCTGCTTCCTCCCC  CCATGGCTGATATCGGATCCATGGCGTCTGCAAACGCTCTC  CAAGCTTGTCGACGGAGCTCACCATGAGACCCTCAGGAGCA |
| ARC3-LP | Genotyping | AAGAAATCTATCCGCTCGAGC |
| ARC3-RP |  | TTGTTTCCGGTCAATGAGAAC |
| CLPC1-401-LP |  | GCTTCCATTGATCTGAGCTAAG |
| CLPC1-401-RP |  | TTGATCTTATCTCTTCAAGGGA |
| PUB52-401-LP |  | AATCGGAGCCTATGGATCAGTC |
| PUB52-401-RP |  | CGGAACAGAGAAAAACACACA |
| proARC3-F | Gene amplification | GGGGTACCAATCAACACAGCCATGAATGAAG |
| proARC3-R |  | CCCAAGCTTCGCTTCTACGGAACCACTTCTTA |
| ARC3-F |  | GGGGACAAGTTTGTACAAAAAAGCAGGCTCCATGCCGATTTCTATGGAACTTC |
| ARC3-R |  | GGGGACCACTTTGTACAAGAAAGCTGGGTAATCTCCGGCGTCCACTTGTTT |
| ARC3-2306-F |  | CGGGGGACGAGCTCGGTACCATGCCGATTTCTATGGAACTTC |
| ARC3-2306-R |  | TGGTCGACTCTAGAGGATCCATCTCCGGCGTCCACTTGTTTC |
| ARC2-2306-F |  | CGGGGGACGAGCTCGGTACCATGGCGTCTGCAAACGCTCTC |
| ARC2-2306-R |  | TGGTCGACTCTAGAGGATCCCACCATGAGACCCTCAGGAGCA |
| PUB52-GWB-F |  | GGGGACAAGTTTGTACAAAAAAGCAGGCTCCATGGAAGAGAAAAAAGTAGTAAGAG |
| PUB52-GWB-R |  | GGGGACCACTTTGTACAAGAAAGCTGGGTACTGGATTGGATGATAAGCCAAG |
| CLPC1-PG-F |  | ACACGATCGATAAGCTTCCCATGGCTATGGCCACAAGGGTG |
| CLPC1-PG-R |  | TCGCCCTTGCTCACCATCCCAGCAACAGGGAGAGAATCTTCC |
| ARC3-4T-BamHI-F | Mutated version | CTGGTTCCGCGTGGATCCATGCCGATTTCTATGGAACTTC |
| ARC3-1R |  | TCCCTCACTAAGCACATCACGCAAATCCGACGCGCCTTTCGAGATGTAGAGTTCA |
| ARC3-2F |  | TGCTTAGTGAGGGATTCTGCTCCGATAGAGACGTCCGAGAG |
| ARC3-2R |  | TAGCCACAATGGATATAATA |
| ARC3-3F |  | TATTATATCCATTGTGGCTA |
| ARC3-R1 |  | ATCGGAGCAGAATCCCTCACTAAGGACATCACGGAAATCCGA |
| ARC3-F2 |  | GTGAGGGATTCTGCTCCGATAGAGACGTCCGAGAGAG |
| ARC3-R2 |  | CGATATCTATAGAGAAATTGGTG |
| ARC3-F3 |  | CACCAATTTCTCTATAGATATCG |
| ARC3-R3 |  | GCCTCCTTGGGATCTTCCCTTGA |
| ARC3-F4 |  | TCAAGGGAAGATCCCAAGGAGGC |
| ARC3-93R1 |  | GAATCCAAGGATGAATCCATG |
| ARC3-93F2 |  | CATGGATTCATCCTTGGATTC |
| ARC3-299R2 |  | GAAAGAATGGGGAGTCATATATAG |
| ARC3-299F3 |  | CTATATATGACTCCCCATTCTTTC |
| ARC3-4T-EcorI-R |  | GAGTCGACCCGGGAATTCATCTCCGGCGTCCACTTGTTTC |
| ARC3-771-F | Split-luc | CGGGATCCATGCCGATTTCTATGGAACTTC |
| ARC3-771-R |  | ACGCGTCGACATCTCCGGCGTCCACTTGTTTC |
| ARC3-772-F |  | CGGGATCCGATGCCGATTTCTATGGAACTTC |
| ARC3-772-R |  | ACGCGTCGACTCAATCTCCGGCGTCCACTTGT |
| ARC2-JW771-F |  | GACGAGCTCGGTACCATGGCGTCTGCAAACGCTCTC |
| ARC2-JW771-R |  | CGAGATCTGGTCGACCACCATGAGACCCTCAGGAGCA |
| ARC2-JW772-F |  | TCCCGGGGCGGTACCATGGCGTCTGCAAACGCTCTC |
| ARC2-JW772-R |  | GCTCTGCAGGTCGACTTACACCATGAGACCCTCAGGAGCA |
| PUB52-772-F |  | TCCCGGGGCGGTACCATGGAAGAGAAAAAAGTAGTAAGAG |
| PUB52-772-R |  | GCTCTGCAGGTCGACCTACCGTTTATTAGATTTCCACTCCA |
| CLPC1-772-F |  | TCCCGGGGCGGTACCATGGCTATGGCCACAAGGGTG |
| CLPC1-772-R |  | GCTCTGCAGGTCGACTTAAGCAACAGGGAGAGAATCTTC |
| SBT1.8-772-F |  | TCCCGGGGCGGTACCATGGCTTCTTCTTCTTCTTCTT |
| SBT1.8-772-R |  | GCTCTGCAGGTCGACTCAAAACCGGTTCCAAGAGAAT |
| FTSH2-772-F |  | TCCCGGGGCGGTACCATGAATTTTCAGATGGCAGCT |
| FTSH2-772-R |  | GCTCTGCAGGTCGACTTAGACAGCAGCTGGTGTTGGT |
| CLPR2-772-F |  | TCCCGGGGCGGTACCATGGCGGTCTCGTTTAATACA |
| CLPR2-772-R |  | GCTCTGCAGGTCGACCTAGCCTAGCCCTGCGCTTTCG |
| CCT3-772-F |  | TCCCGGGGCGGTACCATGCACGCACCGGTACTCGTT |
| CCT3-772-R |  | GCTCTGCAGGTCGACTTAGTCGGGAAGAATTTGTTCG |
| RD21A-772-F |  | TCCCGGGGCGGTACCATGGGGTTCCTTAAGCCAACCA |
| RD21A-772-R |  | GCTCTGCAGGTCGACTTAGGCAATGTTCTTTCTGCCT |
| qARC3-F | Real time PCR | TCGCAAGGAGTCTATCAT |
| qARC3-R |  | AGAACCATATCCAGCACTA |
| qARC2-F |  | TTGAGGATGCTGATGAAC |
| qARC2-R |  | CCAATCGCTGAACATAATC |

**Table S1.** Primers used in this study.
